# A simple and efficient process for the synthesis of 2D carbon nitrides and related materials

**DOI:** 10.1038/s41598-023-39899-5

**Published:** 2023-09-18

**Authors:** Cora Moreira Da Silva, Maxime Vallet, Clément Semion, Thomas Blin, Romuald Saint-Martin, Jocelyne Leroy, Diana Dragoé, François Brisset, Cynthia Gillet, Régis Guillot, Vincent Huc

**Affiliations:** 1 CNRS, Institut de Chimie Moléculaire et des Matériaux d’Orsay, Université Paris-Saclay, 91405 Orsay, France; 2 École Centrale Sup’Élec, Université Paris-Saclay, Paris, France; 3 ONERA, CNRS, Laboratoire d’Étude des Microstructures, Université Paris-Saclay, Châtillon, 92322 France; 4 CEA, CNRS, NIMBE, LICSEN, Université Paris-Saclay, 91191 Gif-sur-Yvette, France; 5 CNRS-Institut de Biologie Intégrative de la Cellule (I2BC), Gif-sur-Yvette, France

**Keywords:** Chemistry, Materials science

## Abstract

We describe here a new process for the synthesis of very high quality 2D Covalent Organic Frameworks (COFs), such a C_2_N and CN carbon nitrides. This process relies on the use of a metallic surface as both a reagent and a support for the coupling of small halogenated building blocks. The conditions of the assembly reaction are chosen so as to leave the inorganic salts by-products on the surface, to further confine the assembly reaction on the surface and increase the quality of the 2D layers. We found that under these conditions, the process directly returns few layers material. The structure/quality of these materials is demonstrated by extensive cross-characterizations at different scales, combining optical microscopy, Scanning Electron Microscopy (SEM)/Transmission Electron Microscopy (TEM) and Energy Dispersive Spectroscopy (EDS). The availability of such very large, high-quality layers of these materials opens interesting perspectives, for example in photochemistry and electronics (intrinsic transport properties, high gap substrate for graphene, etc...).

## Introduction

**Figure 1:**
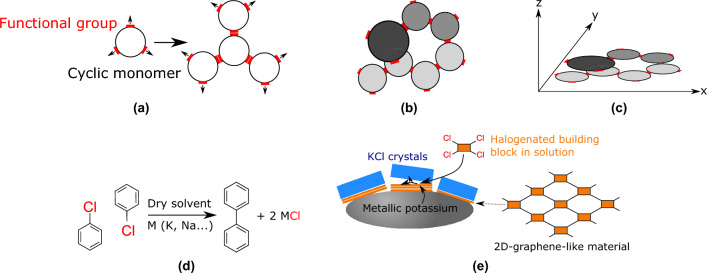
Synthesis strategy schemes. **(a)** General synthesis of 2D materials by building units^24^, **(b)** example of monomer assembly defects, **(c)** example of monomer assembly defects perpendicularly to the average plane of the 2D network, **(d)** Fittig reaction^30^ and **(e)** confining the growth of layered materials by both a surface and a metallic salt.

The first preparation of graphene by Geim and Novoselov in 2004^1,2^ sparked an ever-increasing interest in 2D materials. Indeed, along with graphene, dichalcogenides such as MoS_2_ or WS_2_^3,4^, silicene^5,6^, phosphorene^7–9^, hexagonal boron nitride^10,11^ etc... are also being investigated all over the world, due to their fascinating physical^3,12–14^ and chemical properties^15–17^. More recently, 2D COFs (Covalent Organic Frameworks), a family of 2D materials, has received a growing interest^18–23^. Currently, most (not to say all) the current syntheses of COFs are relying on the assembly of small, multifunctional building units (Fig. 1a, modified/adapted from^24^).

The main difficulty to be tackled here is the occurrence of such defaults as the one shown in Fig. 1b. In this case, the connection between two adjacent units is missed, resulting in the formation of 3D, defective materials. It is thus clear that reducing the degree of freedom of the system along the *z* axis (i.e. perpendicularly to the average plane of 2D network, Fig. 1c) should limit the occurrence of such defaults. A common approach is to adsorb the molecular building units on a surface and condense them by means of different bond-forming reaction^25–29^. Many examples of this strategy are to be found in literature, for example by using reactive Cu surfaces in combination with halogenated aromatics^25^. This strategy returned many beautiful 2D materials but often obtained and studied (e.g. by STM/AFM) under UHV conditions (i.e. impossible to get on a preparative scale). It would thus be interesting to use the same approach under more conventional conditions. An examination of the different metallic surfaces-assisted reactions in solution drives to the conclusion that the well-known Fittig reaction (Fig. 1d)^30^ is ideal.

First, this reaction uses reactive metals (such as potassium or sodium) to promote the formation of a C–C bond between two (aromatic) halides (e.g. chlorides), with the concomitant formation of insoluble Na or K salts as by-products. Second, as neither the starting products nor the final ones are soluble in potassium or sodium, this reaction is thus fundamentally a surfacic process. It is thus an appealing tool for 2D materials. Third, The Fittig reaction is far more reactive than more conventional couplings (e.g Ullman and variations), and there’s no need for organometallic catalysts (Ni or Pd). Last, the starting products are either commercially available or readily synthesized (Fig. 1e).

The by-product of this reaction (KCl in the example shown) is insoluble in the reaction media, and thus precipitates on the surface of the metal. Under these conditions, the reaction may continuously run in a thin layer of liquid sandwiched between the metal and the halide. As the thickness of this layer is automatically reduced by the presence of the upper KCl (or KBr) deposit, two confinements effects may act cooperatively: (i) the involved reaction is intrinsically a surfacic process and (ii) the presence of a KCl or KBr precipitate, restricting the degree of freedom of the growing layers along the *z* axis adds a second confinement effect.

In this work, we wish to propose three examples of this double confinement strategy, that improves the quality of synthetic graphene-like 2D materials.

## Results

Since the pioneering work of Bao et al.^31^ and Chuanbao et al.^32–34^, the synthesis of 2D materials by alkali metal-promoted reduction of halogenated precursors was left largely unexplored. Seeking for simple synthesis, that could be performed using common lab equipment, we designed a protocol that runs under close to ambient conditions. To favor a 2D-confined reaction, the solvent used is chosen so as to dissolve the reactant (i.e. allows for the diffusion of the halogenated species to the metallic surface), but neither the final 2D material, nor the inorganic by-products (or any other metal-containing species), to reinforce the confinement effect.

### Synthesis of CN carbon nitride

**Figure 2:**
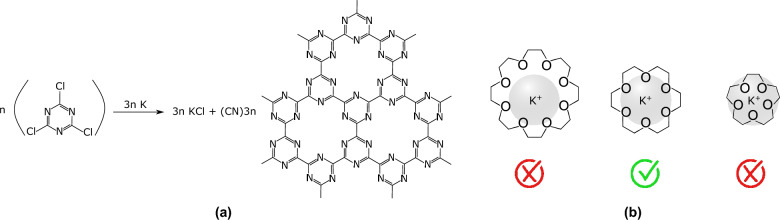
Synthesis of CN carbon nitride. **(a)** Reaction and **(b)** fit of different macrocyclic cavities with the potassium ion^42,43^.

We decided to perform a first experiment targeting a carbon nitride with the general formula CN (Fig. 2a)^32–36^. Indeed, carbon nitrides of various stoichiometries are promising for a plethora of applications^37–41^. Moreover, the envisioned starting product, cyanuric chloride, is a cheap, commercially available one.

Last, potassium was chosen as the reactive metal, because the targeted carbon nitrides contain coordinating cavities with 6 inward-pointing nitrogen ligands^44^. The study of the complexation of K$^+$ ions with various oxygen or nitrogen-containing macrocycles have indeed shown that K$^+$ ions has a high affinity for such cavities, in comparison with other ones with 5 or 8 coordinating atoms (Fig. 2b)^42,43^. It can thus be guessed that this cation will template the selective formation of six-membered cavities, thus further reinforcing the selectivity towards the formation of the targeted carbon nitrides.

The details of the synthetic process are to be found in “Methods” section and in part S1 of the Supporting Informations (SI).

First, an analysis of the crude reaction media (supernatant and solid separately) was performed to qualitatively assess the yield of the reaction. The amount of remaining organics in the supernatant was found negligible (less than 100 mg). The presence of left residual starting product and/or partial reaction products can thus be considered as rather low. Then, a sample of the solid obtained after the filtration step (i.e. the removal of soluble residual organics) was directly analyzed by X-ray Photoelectron Spectroscopy (XPS) (Fig. S1-2 of SI). The Cl 2p spectra can be deconvoluted in two main components, at 197.74 and 199.26 eV. The first one is attributed to KCl (inorganic chloride), and the second one to organic chloride, i.e. to C-bonded chlorine. This later component may be attributed to incompletely reduced species. The relative surfaces of these two components (along with the very low amount of recovered soluble species) drives to the conclusion that the yield of the reaction is over 80%. The deconvolution of the N 1s signals shows two components, at 399.21 and 400.57 eV. The 399.21 eV component is attributed to triazinic nitrogen^45–48^. The higher energy component is attributed to nitrogen bonded to included species, such as metallic cations or H-bonded species. In accordance with the presence of inorganic chloride, strong signals associated with K$^+$ ions are also observed.

**Figure 3:**
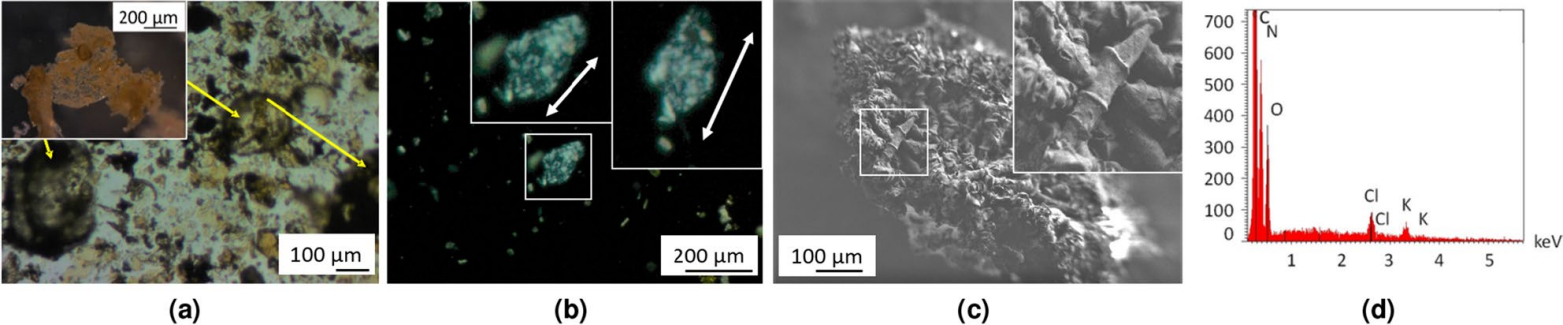
Analyses on CN material. Images acquired by **(a)** optical microscope showing a large and thin flake (zoom on an isolated flake in inset) and hollow spheres (yellow arrows), **(b)** optical microscopy analysis of a flake using polarized light. The brightness of the flake changes with the relative angle between the polarizer and the sample (zoom in inserts, white arrows as a guide to the eye), evidencing its crystallinity). The flake shows different domains due to folds, see S1-3, **(c)** SEM, the zoom in inset shows a rolled up flake. **(d)** EDS analysis on CN sample.

A final washing with water is necessary, to remove the water-soluble KCl by-product. Purified CN is then obtained as a light brown solid, easily redispersed in water. An optical microscopy analysis of a water suspension shows greenish-colored thin and very large flakes (some of them exceeding 500 $\upmu$m) and some hollow spheres, probably the result of a molding of melted potassium droplets (Fig. 3a). These optical microscopy analyses were also performed using polarized light, with flakes suspended in dispersion oil. Note that the observation of other 2D materials such as single layer graphene using polarized light has already been reported^49,50^.

Under these conditions, some flakes appear alternatively bright and dark as a function of the relative angles between the polarizer and the sample, evidence of the crystallinity of the material (Fig. 3b). The fact that different domains appear inside the flake is attributed to folds. Other flakes appear as uniformly bright, regardless of the angle. This could be attributed to the fact that they are constituted of several stacked layers, without orientational correlations between them. The presence of extensive crumpling may also be a plausible explanation, as these folds may also change the polarization angle of the transmitted light (see Fig. S1-3 of SI). More images, other examples of polarized light analysis and handling experiment are provided in the SI (Fig. S1-3).

The presence of large CN flakes is also confirmed by SEM. It shows that they tend to roll (Fig. 3c). These features confirm the formation of a layered material. An EDS analysis shows the presence of the expected elements, along with residual KCl (Fig. 3d). Oxygen is also observed, probably as trapped/adsorbed species.

**Figure 4:**
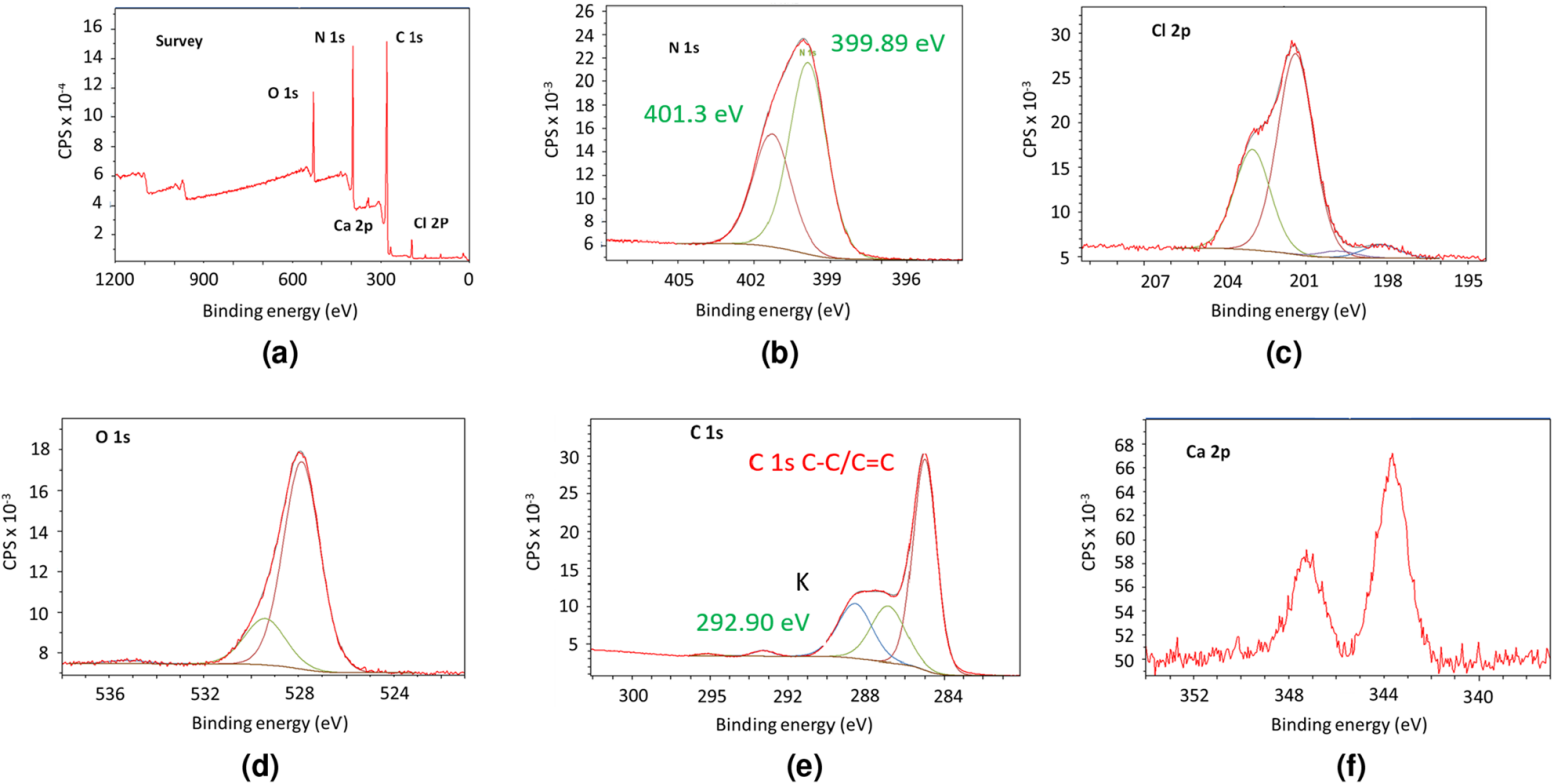
XPS spectra of CN. Signals of **(a)** survey, **(b)** nitrogen, **(c)** chlorine, **(d)** oxygen, **(e)** carbon and **(f)** calcium.

To confirm, an XPS analysis of a washed sample of CN is shown in Fig. 4. Analysis of the Cl 2p core-level spectrum revealed the presence of two spin-orbit doublets whose Cl 2p$^{3/2}$ components are located at 201.3 eV and 198.2 eV. The higher binding energy (BE) component is attributed to traces of residual C–Cl bonds while the lower BE component may be related to chloride ions, as remaining KCl and CaCl_2_. The very low intensities of the C–Cl signal confirm the low amount of defaults in the material. The deconvolution of C 1s signals shows three main components. The first one at 285 eV corresponds to carbon contamination, ubiquitously encountered in such analyses. The two other main components can be attributed to triazinic carbons. The presence of these two signals may result from the interaction of some triazinic N-atoms with guest species (such as metallic cations or H-bonded, adsorbed species). This would in turn increase the polarity of the C=N bonds and thus increase the 1s binding energy of the corresponding carbons.

Regarding the N 1s signal, two main components are observed at 399.91 and 401.35 eV^45–48^. The high energy component has strongly decreased compared with the crude sample of CN. This confirms the attribution of this high energy peak to nitrogen interacting with included species, such as metallic cations. This may also be the result of partial protonation of pyridine nitrogen, as concluded from the IR study (see below). Oxygen is still observed in the material, showing the presence of included, oxygen-containing species. Such a tendency to absorb invited species is not surprising, considering the open structure nature of this material. Similar effects have already been observed with carbon-related materials^51–54^. The C/N intensity ratio between normalized 1s peaks from the relevant carbon signals (*i.e.* those with the highest BE, belonging to C=N bonds) and nitrogen is 1.2, close to the expected value of 1.

An IR spectra of CN compared with reference ones of triazine and starting cyanuric chloride are provided in Fig. S1-4 of SI.

It first shows the absence of signals associated with C–H bonds, thus excluding the existence of defaults in the structure that may have resulted from over reduction of CN. This also confirms the absence of significant amounts of contamination (e.g. residual solvents), systematically exhibiting strong signals in this wavenumber range. Second, the characteristic vibrations of the triazine aromatic ring are observed, confirming the proposed structure of the CN COF^55^. Signals are also observed around 2100 cm$^{-1}$, that could be ascribed to protonated nitrogen species^56^. The presence of these protonated species may explain the presence of high energy component in the XPS spectra of both N and C. A comparison between CN and the starting cyanuric chloride shows the complete disappearance of the otherwise strong signal associated with the C–Cl bond at 850 cm$^{-1}$. This confirms the conclusions returned by XPS, indicating the absence of significant amounts of chlorine in CN.

**Figure 5:**
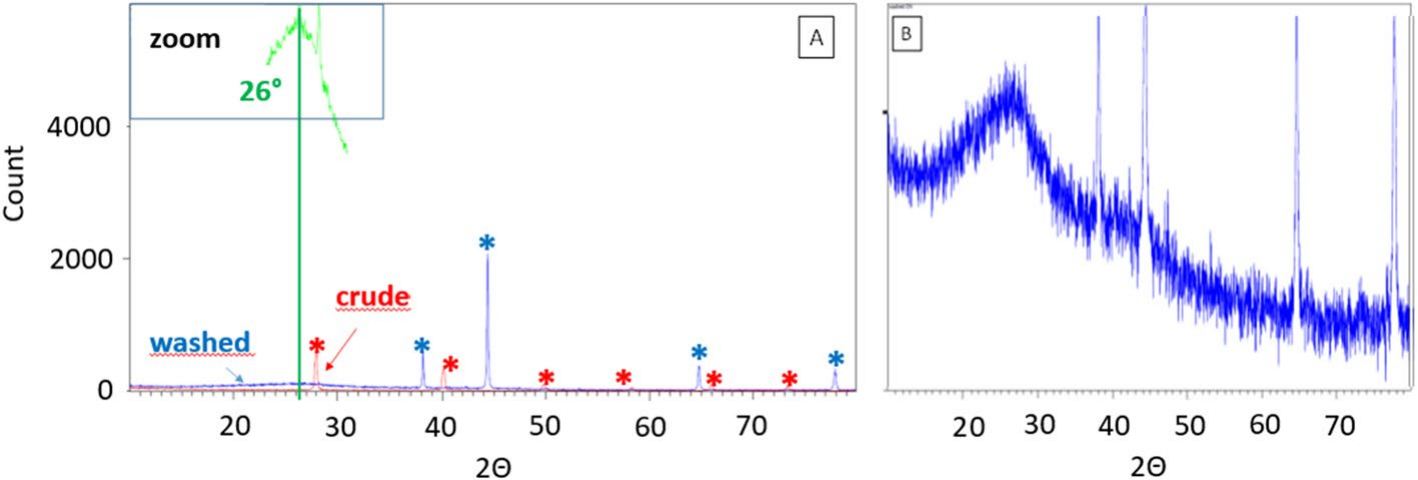
Powder X-ray diffractograms. **(a)** A crude (red) and washed (blue) CN samples (same batch). Insert (green): zoom over the blue curve. **(b)** A washed CN sample: signals from the aluminum sample holder (blue stars) and signals from KCl by-product (red stars).

To investigate the crystallinity of the material, a compared X-Ray Diffraction (XRD) analysis of a crude and washed CN sample was performed. The crude sample only shows signals from the by-product of the reaction, i.e. KCl (Fig. 5A, in red). No others peaks are visible. Interestingly, strong evolutions of the same sample are observed upon washing (Fig. 5A, in blue). Along with the disappearance of the KCl-related signals, a broad peak, centered at 26$^{\circ }$ appears (Fig. 5A, green insert and Fig. 5B), that was absent from the crude sample. As this peak is characteristic of the interplane spacing of graphenic materials, it can thus be concluded that the flakes mostly exist as individual/few layers objects in the crude material. These flakes are probably kept apart inside a KCl matrix. Upon washing and removal of KCl, these flakes then get aggregated, resulting in the appearance of the 26$^{\circ }$ peak. Another conclusion that is to be drawn from this comparative study is that the 2D layers are likely to grow as single/few layers aggregates during the synthesis.

**Figure 6:**
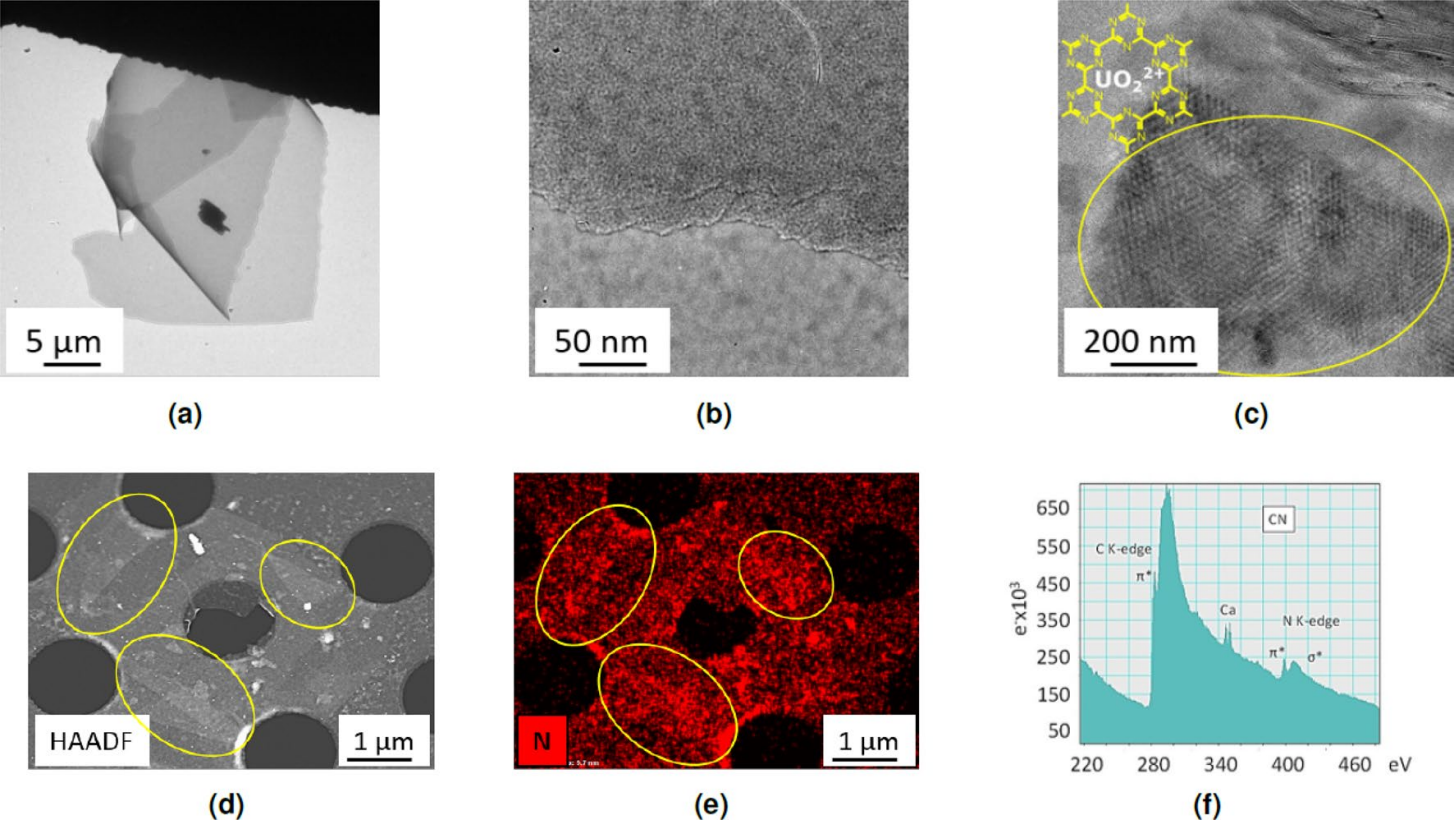
TEM analyses on CN-washed material. **(a)** TEM image in bright-field mode at low magnification showing large flake, **(b)** TEM image in bright-field mode at high magnification showing homogeneous, thin and layered flake, **(c)** TEM image of isolated uranyl ions loaded flake observed at 120 kV showing Moiré pattern, **(d)** HAADF-STEM image of CN flakes deposited on holey carbon grid showing thin flakes with folds (yellow circle), **(e)** STEM-EDS mapping mode of nitrogen (K$\alpha$ line) in red, **(f)** EELS spectrum.

For a more in-depth analysis, different techniques accessible through transmission electron microscopy (TEM) were used on the washed CN material. First, the images are showing the quality of the flakes over different scales (Fig. 6a–c). The material appears as constituted of very large flakes, in accordance with optical microscopy and SEM images. These flakes are very homogeneous and thin, most of them seemingly constituted of bilayers (Fig. 6b). They appear quite smooth, with very few visible defects. The well-known affinity of planar hexacoordinating ligands for uranyl ions was used to load CN with this ion (to increase contrasts). A sample of CN was loaded with uranyl acetate (Fig. S1-5 of SI). We first check the presence of the uranyl cations by XPS analysis (Fig. S1-5a). The observed N/U atomic ratio is around 6, very close to the expected value for the inclusion of uranyl cations in all the hexacoordinating cavities of CN. Note that remaining KCl is also observed (as chloride and K$^+$ ions). An XPS analysis of the same sample was realised after extensively washings it with water (Fig. S1-5b). Whereas the XPS signals of chlorine and potassium have completely disappeared, the N/U atomic ratio was found to be unchanged (around 6), evidencing the strength of the complexation of uranyl cations by the CN carbon nitride. This by itself constitute an indirect but strong confirmation of the structure of CN. The TEM images obtained on an isolated, uranyl-loaded flake show the presence of Moiré effects (i.e. interference pattern resulting from the superposition of two or more networks) from superimposed layers, thus evidencing their crystallinity (Fig. 6c). Indeed, such Moiré effects can only be observed with crystalline materials. Note that this effect also supports the conclusions driven from XPS experiments: an incomplete, random occupancy of the coordinating sites by uranyl cations would have broken the regularity of the network, thus preventing the observation of any Moiré. Other images are provided in the Supporting Informations, also evidencing the layered structure of the material (Fig. S1-5c).

High-angle annular dark-field imaging in scanning transmission electron microscope mode (HAADF-STEM) image shows the presence of very thin flakes (Fig. 6d). A comparison between Fig. 6d and the corresponding nitrogen mapping (Fig. 6e) shows a correlation between the shape of the flake and the intensity of the N signal. Moreover, the intensity of this signal increases where thicker flakes are observed (folds, yellow circles). These observations are showing that the flakes are made of a N-rich material, as expected. This EDS analysis also shows the presence of K and Ca (Fig. S1-6), in accordance with the previous analyses. An electron energy loss spectroscopy (EELS) study of this material shows the presence of the expected elements C and N (Fig. 6f). Ca is also observed as an impurity from the starting metallic potassium. The structure of the C 1s and N 1s edges show a fine structure that is characteristic of extended $\pi$-delocalization^57^, in accordance with the expected structure of CN.

All these analyses show the presence of residual metals (K and Ca, Fig. S1-6 and 6f) in the material, most probably as cations included inside the coordinating cavities of CN. This may explain the presence of the different signals observed for N 1s by XPS analysis. Indeed, the direct coordination of the N to the positively charged cations probably gives rise to the observed higher energy component.

### Synthesis generalization

**Figure 7:**
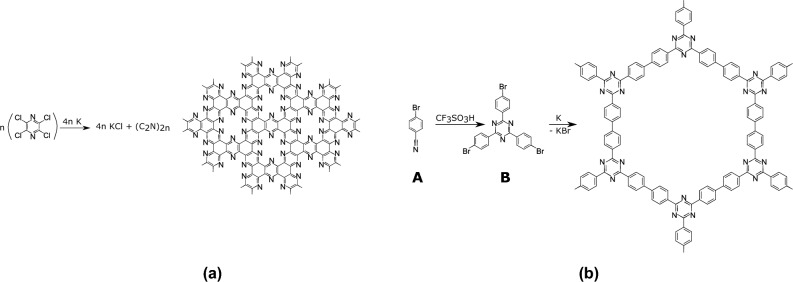
Synthesis of (**a**) C$_2$N and (**b**) 2D material by reduction of a polyhalogenated triazine derivative. The starting tris(bromophenyl) triazine B was synthesized by acid-promoted trimerisation of 4-(bromo)benzonitrile A^58,59^.

These very encouraging results prompted us to generalize our synthetic strategy to C$_2$N carbon nitride and triazine-based COF (Fig. 7).

#### Synthesis of C$_2$N carbon nitride

As a second example, we have chosen to synthesize a well-documented 2D material, the C$_2$N carbon nitride (Fig. 7a). Since its discovery in 2015^51^, this material is under the spotlight, due to its tremendous properties in many different fields^52,60–69^. Only a handful of synthetic approaches are described so far, most relying on the condensation of complementary carbonyl and amine containing reagents under different conditions^64–66,70^, or high temperature pyrolysis of organic precursors^53^. In our work, the targeted carbon nitride is obtained by reduction of tetrachloropyrazine using potassium. The details of the synthetic process are to be found in the “Methods” section and in part S2 of SI.

The reaction media was filtered and the resulting black solid washed with acetone. The filtrates were combined and evaporated to dryness on a vacuum line, leaving a very small amount of a dark brown residue (50 mg, to be compared with about 1 g of starting tetrachloropyrazine).

An XPS analysis of the complementary precipitate (Fig. S2-2) shows the presence of strong potassium signals as cationic species. Cl 2p signals show the presence of two different kinds of chlorine atoms. The first one, with the lowest binding energy is characteristic of anionic chlorine, ascribed to KCl and confirmed by the measured 1/1 K/Cl ratio. The second, with a higher BE, is attributed to organic chlorine (i.e. bonded to carbon atoms). A comparison of the relative areas of these two species shows that the yield of the reaction is above 90%. N 1s signals reveals two components attributed to pyridinic-type nitrogen^45–48^. The higher energy one probably belongs to metallic cations-bonded nitrogen. As in the case of CN carbon nitride, strong signals of cationic potassium (along with a few percent of calcium) are also observed (Fig. S2-2d).

**Figure 8:**
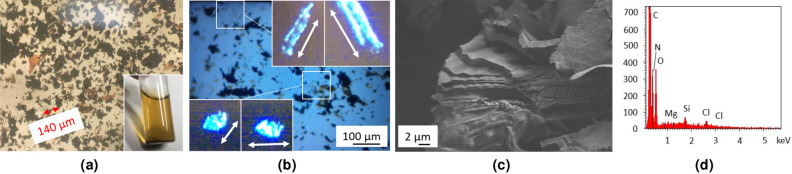
Analyses on C$_2$ N material. Images acquired by **(a)** optical microscope showing black, large, and thin flakes (in inset orange-colored HCl suspension of C$_2$N), **(b)** optical microscope in polarized light mode (the brightness of the flake with the angle (white arrows as a guide to the eye), evidencing its crystallinity), in inset zooms on flakes at different relative angles between the polarizer and the sample showing different domains due to folds, **(c)** SEM. **(d)** EDS analysis.

After a final washing step with water (see “Methods” section), the purified C$_2$N material is obtained as a fluffy, black powder. An observation of the material as an acidic (1 M HCl) water suspension shows it to be constituted of large, variously aggregated and folded flakes, with sizes ranging from a few tens to 300 $\upmu$m (Fig. 8a). The color of the thinnest flakes appears as deep orange, rapidly turning to black with increasing thickness. The bright orange color of acidic C$_2$N suspensions confirms that a significant amount of the material is made of single/few layers flakes. In some instances, tubular or shell-shaped objects were also observed. In the latter case, this is likely to be the result of a molding of melted potassium droplets, getting encapsulated during the growth of C$_2$N around them (Fig. S2-3). Interestingly, we also found that some flakes (thin enough and not too folded/crumpled) do rotate the polarization plane of incident polarized light, as expected for single crystals (Fig. 8b). These extremely thin flakes are nearly invisible (even with the microscope). They can only be detected due to their changing brightness under polarized light. As the flakes gets thicker, the effect disappears, probably due to (i) the absence of correlation of the orientation of the layers inside thick flakes, (ii) their increasing absorbance and/or (iii) the presence of folds, that tend to average the effect on polarized light.

More images and other examples of polarized light analysis are provided in the SI (Fig. S2-3).

SEM observation confirms the presence of large and layered flakes ($>10~\upmu$m) (Fig. 8c), in accordance with the expected one for C$_2$N^51^, and EDS analysis shows the presence of the expected elements, along with SiO$_2$ from the substrate (Fig. 8d).

XPS analysis (Fig. S2-4) shows a prominent oxygen peak, may be due to the presence of trapped oxygen-containing species, a commonly observed feature with this type of material^51–53,70^. The deconvolution of the C 1s peak shows two main components, at 285.9 eV (C=N) and 284.9 eV (C–C). This later peak, ubiquitous, may originate from contamination. The former one is associated with the C=N bonds of the 2D framework. Minor peaks at higher binding energies are from C=O containing adsorbed contamination, commonly encountered in XPS analyses. These may also originate from the interaction between N and included species (protons, as the material was washed with HCl, or remaining metallic cations), in line with the observed features of the N 1s signals (see below). Indeed, the influence of these trapped species may affect as well both nitrogen and the adjacent, directly N-bonded carbon atoms. Regarding the N 1s signal, it may be deconvoluted in two components, at 399.12 and 400.46 eV. The first one is attributed to pyridinic-type N, as discussed in the previous paragraph^45–48^. Indeed, our synthesis does not involve any kind of NH$_2$-type species (that appear in the same binding energy range), thus ruling out this possible attribution. Moreover, the dark black appearance of our material excludes any kind of reduction reaction (that could result in the formation of N–H species), that would break the conjugation of the network, rendering the material colorless. IR spectroscopy also rules out this possibility (see discussion below). Moreover, tetrachloropyrazine was introduced in close to stoichiometric amount vs. potassium. It thus excludes a possible over reduction of the C$_2$N backbone to N–H containing species, as this would need significant amounts of excess metal. Moreover, as the post-synthesis workup was done under ambient conditions, such adventitious reduced species would have been re-oxidized to the main C=N ones (especially under the acidic conditions used for the redispersion of the material). The high energy pyridinic component (400.46 eV) may be attributed at first sight to residual tetrachloropyrazine. However, this interpretation is ruled out, as the amount of remaining C–Cl in the product is very low. This interpretation also seems unlikely for another reason: soluble organics (unreacted starting pyrazine or by-products) are recovered in the supernatant and acetone washing filtrates and the corresponding amount was found to be very low. This high energy component is thus better attributed to the interaction of pyridinic nitrogen with included, remaining metallic cations or hydrogen-bonded impurities that appeared during post-synthesis workup. Indeed, it was shown that the exact value of the position of the N 1s peak is strongly dependent on the environment of the corresponding nitrogen^45–48^. This last interpretation is in line with the presence of oxygenated species and remaining K and Ca. The C/N ratio is 3, higher than the expected value of 2. This may be ascribed to commonly observed carbon contamination of the sample. As said before, traces of chlorine are also observed, with binding energies associated with C-bonded chlorine. This confirms the very small amount of defaults lefts in the flakes, as only a very small amount of unreacted pyrazinic C–Cl bonds is present in the material.

As it is the case for CN, the presence of hexacoordinating cavities in C$_2$N makes it suitable for the complexation of uranyl cations. An uranyl-loaded C$_2$N sample was subjected to XPS analysis. The results are shown in the Supporting Informations (Fig. S2-10). Two different places were analysed, and the N/U atomic ratio was found equal to 6 in both cases, as expected. This constitutes an indirect but strong confirmation of the structure of C$_2$N, as already explained in the case of CN. To confirm, IR spectra of C$_2$N was compared with two reference compounds: starting tetrachloropyrazine on the one hand, and the building unit pyrazine on the other hand (Fig. S2-5). As observed for CN, the absence of C–H peaks for C$_2$N rules out the presence of (i) structural defaults resulting from over reductions, and (ii) significant amounts of contaminating species. Second, signals attributed to characteristic vibrational modes of the pyrazine ring are observed (stretching C=C and C=N along ring deformation), confirming the proposed structure of C$_2$N^55^. As observed for CN, signals are also observed around 2100 cm$^{-1}$. These peaks may correspond to protonated, pyridinium–nitrogen, that are usually observed in this wavenumber area^56^. The IR spectrum of the starting tetrachloropyrazine shows a very strong C–Cl peak at 655 cm$^{-1}$, absent in C$_2$N. This confirms the success of the assembly reaction and reinforces the conclusions drawn from XPS analysis, regarding the absence of chlorine in our material. A Raman analysis of a single C$_2$N flake is provided in the SI (Fig. S2-6). It shows very intense peaks, corresponding to C=N and C=C bond stretching vibrations, as expected. No signals associated with C–H bonds are observed, thus confirming the conclusions drawn from IR studies.

A compared XRD analysis of a crude and washed C$_2$N sample was performed and the same trends as those observed with CN are evidenced (Fig. S2-7). The crude sample only shows signals from the KCl by-product. These peaks are no longer observed on the same sample, after washings. Its main feature is a broad peak centered at 27$^{\circ }$, indicating a layered structure. The broadness of the peak evidences an irregular spacing between the sheets. Indeed, these observations can be rationalized by the growth of C$_2$N as single/few layers, getting aggregated during washings. Folds and/or inclusion of residual solvents between the layers occurs during this process, resulting in a broad distribution of interplane spacings. A shoulder around $2 \Theta =40^{\circ }$ corresponds to the in-plane signal^52,53^.

**Figure 9:**
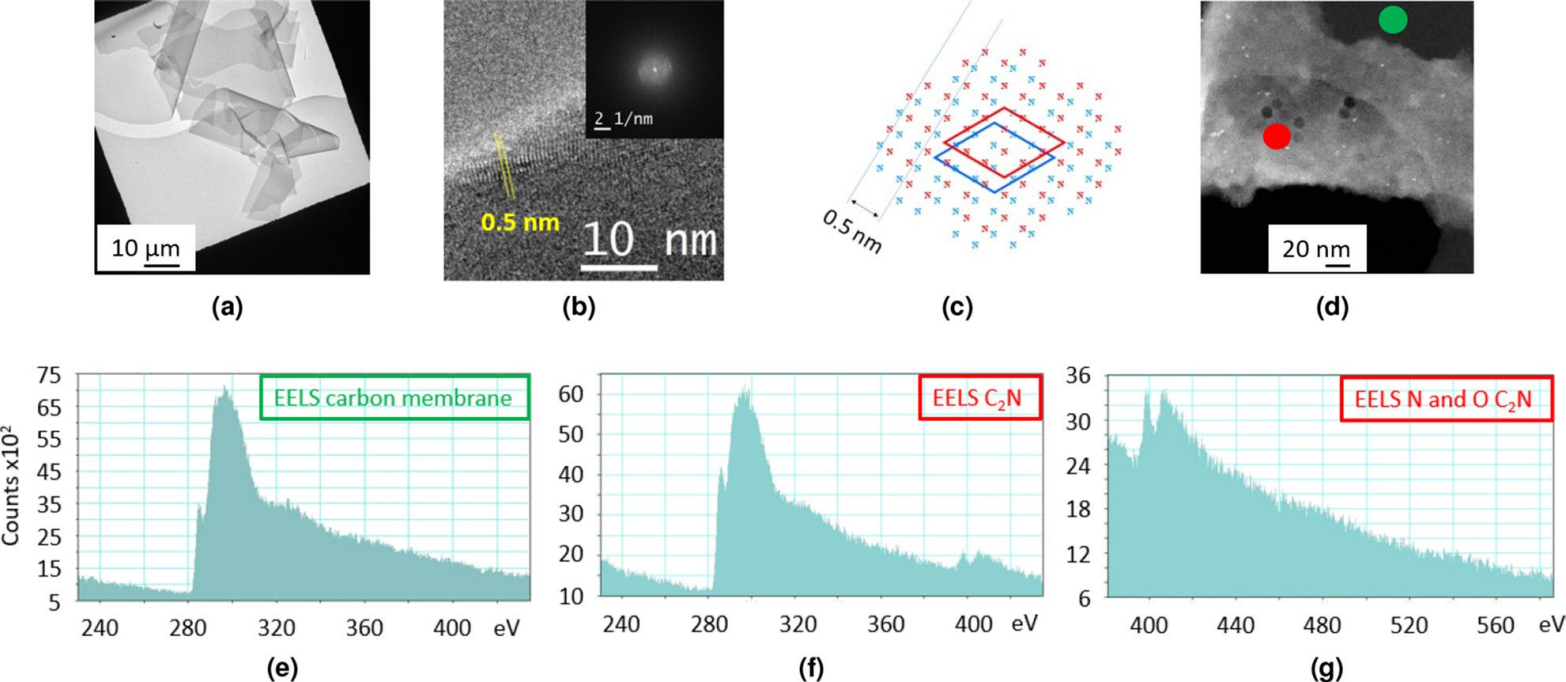
TEM analyses of C$_2$N. **(a)** TEM image at low magnification showing large, thin and homogeneous flakes. **(b)** HRTEM on a flake showing atomic rows (distance = 0.5 nm) and FFT of the image in inset. **(c)** Two stacked C$_2$N layers (one layer in red and the other in blue, only nitrogen atoms shown for clarity, unit cells of the two stacked layers shown as rhombus) exhibiting the observed 0.5 nm distance. **(d)** EELS analysis of a single flake suspended on holey carbon grid*: **(e)** carbon grid analysis (green circle on the image (**d**)), **(f,g)** C$_2$N sample (red circle on the image (**d**)). *The regular, circular holes observed on the sample are due to the electron-beam-induced damages.

Extensive TEM analyses were performed on washed samples. Different solvents were tested to optimize the dispersions (see SI, Fig. S2-8). HCl was shown to be the best choice for the redispersion of the different materials, in accordance with previous observations^71^. Very large, smooth and defects-free flakes are observed, with sizes exceeding 100 $\upmu \hbox {m}$, as previously observed for CN (Fig. 9a). Some flakes are so large that they were first misinterpreted as broken carbon films from the TEM grid. However, a detailed analysis of the micrographs showed that (i) some typical features of carbon grids next to the flakes are still observed, and (ii) the contrasts next to different flakes on different squares were found similar, thus proving that the carbon film was still present. The flakes are rather thin, being quite transparent to the electron beam. A detailed observation of the edges of the flakes shows a regular pattern, exhibiting a characteristic distance around 0.5 nm (Fig. 9b). This pattern is attributed to a Moiré effect between few superimposed layers. One possible matching arrangement is illustrated in Fig. 9c, with two superimposed layers with an AB type stacking. Note that this proposed arrangement is quite similar to the one expected from simulations^72^. This Moiré effect evidences the crystallinity of the whole flake, as there’s no reason for it to be more crystalline at its edges. We were not able to get convincing electron diffractions patterns from this material. This may be due to the instability of the material under electron irradiation^73^. In some instances, clear diffraction patterns were observed (examples shown on Fig. S2-9). However, these were finally considered as artifacts as (i) the characteristic distance associated with the [100] plane was not observed, and (ii) the brightness of the spots was too high to be associated with few layers flake. These artifacts may be due to remaining inorganics trapped inside the material. Great care should thus be taken while analyzing the selected area electron diffraction (SAED) of layered 2D materials.

An EELS analysis of a C$_2$N thin flake only shows nitrogen on the flake, not on the carbon grid, and C$_2$N appears as oxygen-free Fig. 9d–g. This confirms that the oxygen signals observed by XPS analysis are probably due to contamination, or to adventitious oxygen-containing species trapped between aggregated flakes. The near edge structures observed for the C and N signals confirms that these elements are sp$^2$ hybridized in the material^74^.

#### Synthesis of a triazine-based COF

The potassium-promoted reduction of polyhalogenated aromatic building blocks thus appears as a very efficient tool for 2D materials synthesis. We then decided to further expand the scope of this process threw a third example, sketched on Fig. 7b. This material belongs to the so-called Covalent Triazine Frameworks (CTF) family. These materials are indeed receiving an ever increasing interest, due to their interesting properties for example in the field of photocatalysis^75–77^.

The details of the synthetic process are to be found in “Methods” section and in part S3 of SI.

**Figure 10:**
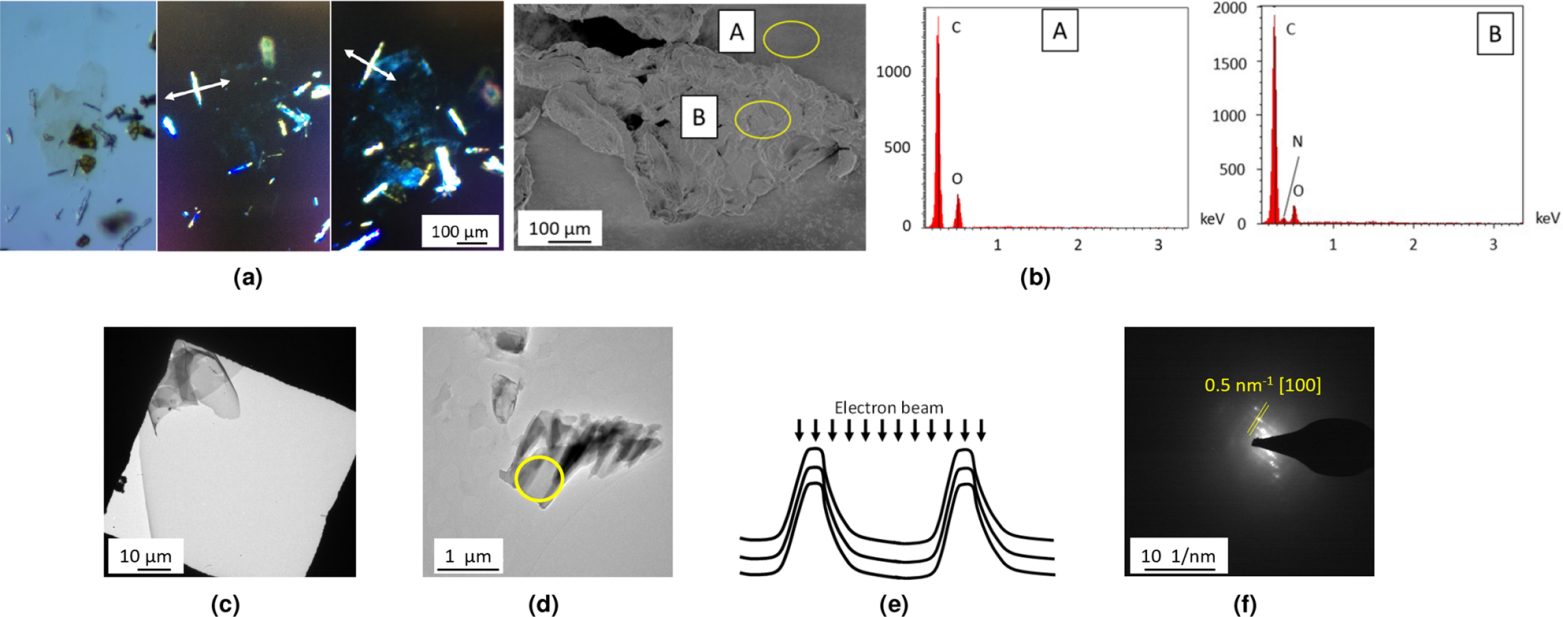
Analyses of CTF-2D material. **(a)** Optical microscopy at normal (1) and in polarized light ((2) and (3)) at different relative angles (white arrows as a guide to the eye) showing large, thin, folded and crystalline flakes. **(b)** SEM image of the sample and EDS analysis on points A and B (yellow circles), **(c)** TEM images confirming previous observations. **(d)** TEM image of folded flake (yellow circle: SAED analyzed area), **(e)** model of the analyzed area and **(f)** corresponding SAED analysis.

Interestingly, a detailed observation of the surface of potassium during the reaction shows that CTF grows in a bark-tree like fashion, along with KBr scales, both visible with the naked eye. This concomitant formation of layered KBr/CTF strongly supports our initial assumption that the inorganic by-product (KBr in this case) is likely to favor the confined growth of 2D materials. CTF material is obtained as a green–brown powder after drying, washing with dichloromethane and water. An observation of this material (as a water suspension) by optical microscopy shows it to be made of very large, thin flakes, with sizes ranging from a few to 500 $\upmu$m. The characteristic features of 2D materials (such as folds) are easily observed. The flakes appear as nearly transparent, evidencing their small thickness and large bandgap. Their yellow-green color is quite similar to the one observed using a different synthesis of this material^75^. Needle-like crystals of the starting material are sometimes observed, as this starting tris(bromophenyl)triazine is highly insoluble. As for the previously described materials, we also found that some flakes (thin enough and not too crumpled), suspended in dispersion oil do rotate the polarization plane of incident polarized light, as expected for single crystals (Figs. 10a, S3-2).

SEM image of CTF-2D material confirms the presence of large, folded flakes and EDS analysis of such a flake shows the presence of C, N and oxygen-containing contamination (Fig. 10b). Note that the N signal is absent from the carbon substrate used for this experiment. The low relative intensity of the N signal is ascribed to (i) the low intrinsic relative amount of nitrogen in the material (C/N$_{theo}=7$), and (ii) the presence of the underlying carbon substrate. In the same way, an EDS analysis of the needle-shaped objects sometimes observed with the CTF flakes shows the presence of bromine along with C, N and O. This confirms the attribution of these needle-shaped objects to the starting product (Fig. S3-3). A comparison between the EDS analyses of CTF-2D material and the starting product shows that the relative C/N ratio is close in both cases, as expected.

An XPS analysis of CTF-2D material shows the presence of strong signals from oxygen and silicon (with a relative O/Si intensity=2), from the silicon wafer used as the substrate for the analysis (Fig. S3-4). All the expected elements are observed, essentially carbon and nitrogen, with a relative C/N intensity of 12, the expected value being 7. This excess carbon is likely to be due to commonly encountered contamination. Only traces of bromine are observed, coming from the remaining unreacted starting product.

An IR spectra of CTF is provided in the SI (Fig. S3-5), and compared with a reference one of the starting tribromotriazine derivative. The signals attributed to the characteristic vibrational modes of the triazine and phenyl rings are observed (stretching C=C and C=N and ring deformation), in the same wavenumber range compared with the starting product. This confirms the proposed structure of CTF.

Another interesting feature is the complete disappearance of the strong signal at 540 cm$^{-1}$, attributed to the C-Br bond, on going from tribromotriazine to CTF. It confirms the occurrence of the expected coupling reaction, and thus the structure of the product. It also confirms the conclusions drawn from XPS experiments.

A Raman analysis of a single CTF flake is provided in the SI (Fig. S3-6). It shows the presence of a broad peak around 1600 cm$^{-1}$, corresponding to C=N and C=C bond stretching vibrations, as expected. No signals associated with C−H bonds are observed, thus confirming the conclusions drawn from IR studies.

As for the two previous materials, TEM images confirm very thin and large flakes, with length sometimes exceeding 100 $\upmu$m (Fig. 10c). The flakes appear as very homogeneous and seemingly defect-free, even at large magnifications. In a few places, some contamination by what seems to be needle-shaped crystals of the starting product are however observed, in accordance with optical and EDS observations. Some rare, highly folded CTF flakes were sometimes observed. A careful examination of the corresponding high resolution TEM micrograph over folds allows to get a rough estimate of the thickness of the flakes (Fig. S3-7). The obtained values fall between 1.5 and 3 nm, corresponding to 5 to 10 2D layers. Some rare tube-shaped objects were also observed, reminiscent of a bamboo-like growth of carbon nanotubes^78^. This may be due to a molding of potassium droplets during the growth. SAED pattern obtained from the flake shows the characteristic distance of 2.2 nm expected for the [100] plane of TPTZ (Fig. 10d–f). In the same way, a distance of 6 Å along the *z* axis is observed (corresponding to the thickness of two conjugated layers) indicative of an AB type stacking. The observed SAED diffraction pattern is characteristic of several crystalline, stacked layers oriented parallel to the incident electron beam^79^. It is thus likely that in the present case, the observed pattern originates from the folded sides of the studied area. It was not possible to observe diffraction on flat-lying sheets, presumably due to their small thickness and the very porous structure of CTF-2D layers. This experiment confirms that the CTF flakes are crystalline. As for the two previous 2D materials, artifacts were commonly observed during SAED experiments. They were finally discarded after careful analyses, as the characteristic distances expected for the [100] plane were never observed (Fig. S3-7). These artifacts may originate from trapped inorganic salt, or remaining starting product.

### Synthesis of C$_2$N using mercury/potassium amalgam

In line with the *double confinement* strategy, the synthesis of the C$_2$N carbon nitride was also realized using another kind of reactive metal interface, the mercury/potassium amalgam. Indeed, the propensity of mercury to form liquid alloys with many metals, including alkaline ones is well known. We reasoned that under these conditions, the metallic interface should be more stable, as mercury itself is not reactive towards tetrachloropyrazine. This could in turn favor the formation of even higher quality 2D layers. Moreover, the liquid nature of the mercury/potassium amalgam should allow for a continuous supply of metallic potassium to the organic solvent/metal interface thus compensating for its consumption during the formation of the C$_2$N 2D network. Last, the inorganic by-product (KCl) is completely insoluble in the solvent used for the reaction (toluene), thus increasing the confinement effect.

The details of the synthetic process are to be found in “Methods” section and in part S4 of SI.

**Figure 11:**
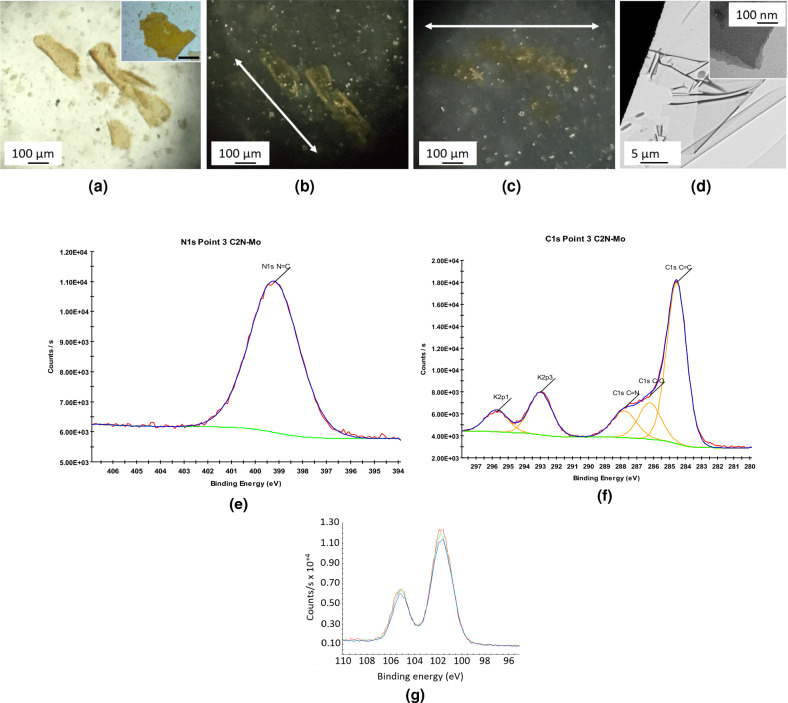
Analyses of C$_2$N material using mercury/potassium amalgam suspended in a 1:2 EtOH:HCl 2 M (v/v) solution. Optical microscopy at **(a)** normal light (in inset photography of large C$_2$N flake, scale bar = 300 $\upmu$m) and **(b,c)** polarized light at different relative angles showing large, thin, folded and crystalline flakes. **(d)** TEM image showing large, thin, layered (in inset) and homogeneous flakes. XPS analyses with spectra of **(e)** nitrogen, **(f)** carbon and potassium and **(g)** evolution of Hg at the same place as a function of time.

The initially shiny mercury interface rapidly gets covered with an orange-greenish deposit. The amalgam is removed with a pipette, and the solid supernatant is washed with ethanol and water. The material is obtained as a light brown/orange powder, with many orange scales easily visible with the naked eye (Fig. 11a).

Optical microscopy analysis shows the presence of very thin (transparent), very large flakes (often mm sized)(Fig. S4-2), all exhibiting the characteristic orange color shades previously observed for thin C$_2$N flakes. This confirms that the mercury amalgam-based synthesis indeed mostly results in single/few layers flakes. Only a small amount of black materials (strongly aggregated/folded flakes) is observed.

A polarized light analysis of these flakes shows them to be quite crystalline, as they alternatively appear bright/dark as a function of the angle between the polarizers (Figs. 11a–c, S4-3). If kept in suspension for several days, the flakes tend to fold and roll, but are still crystalline.

In line with the previous C$_2$N synthesis, very large, smooth and layered flakes with few visible defects are observed by TEM, even at high magnifications (Fig. 11d). An examination of the edges of these flakes clearly shows their layered structure. In order to insure that the observed objects are not artifacts (broken carbon film from the grid), a TEM analysis of the same sample was performed using a holey carbon grid (Fig. S4-4). It indeed confirms the presence of very large flakes. Some expands over several squares on the TEM grid. All are showing the characteristic features of sheet-like materials (folds, overlaps, etc...). Some rare, highly folded C$_2$N flakes were sometimes observed. A careful examination of the corresponding high resolution TEM micrograph (two examples in Fig. S4-4) over folds allows to get a rough estimate of the thickness of the flakes. The obtained values fall between 4 and 9 nm, corresponding to 10 to 20 2D layers.

A XPS analysis of one single C$_2$N flake shows the presence of strong signals from oxygen and silicon (with an O/Si atomic ratio of 2), from the native SiO$_2$ layer of the silicon wafer (Fig. 11e–g). Carbon and nitrogen are observed as expected. Regarding the N 1*s* signal, only one single peak is observed, with a binding energy characteristic of pyridinic-type nitrogen atoms^45–48^. The observed relative C/N intensity is 3, higher that the expected value of 2. This is likely to be due to commonly encountered carbon contamination. This is confirmed by the presence of a strong signal around 285 eV on the C 1*s* spectra. This C/N ratio is the same as the one measured for the previously described synthesis of C$_2$N (using potassium only as a reducing agent). Remainings of mercury (1 atomic%) and potassium (3 atomic%) are also observed, due to left starting products/by products. Chlorine is also observed as chloride ions, in a close to 1-to-1 atomic ratio vs. potassium, indicative of the presence of remaining KCl. This confirms the absence of significant amounts of left C–Cl bonds, and thus the formation of a continuous, defaults-free C$_2$N network. The signal of mercury steadily decreases during vacuuming, confirming its presence as a metal.

## Discussion

The structure and composition of all three COFs described in this work are assessed by extensive cross-analyses (optical microscopy/XPS/XRD/SEM/TEM/EDS). The conclusions are as follows:

1. The presence of high amounts of potassium halides in the crude materials (that are completely removed upon washing with water), as expected if the Fittig coupling reaction occurred (Fig. 1).
2. The nearly complete absence of halogens in our final, (washed) materials i.e. the (quasi) absence of remaining C–Cl or C–Br bonds confirms that the assembly reaction occurs cleanly.
3. The presence of strong signals associated with pyridinic-type nitrogen in all our materials as evidenced by XPS.
4. EELS experiments confirm point 3, as observed C and N signals are typical of species belonging to conjugated structures.
5. The crystallinity of our materials, confirmed by TEM (with SAED for one them) and optical microscopy experiments.
6. The compositions are very similar to literature-reported ones.
7. Infrared spectroscopy shows the presence of their expected, respective constituting units for all three COFs.

Taken together, all these conclusions clearly demonstrate the success of our synthetic strategy.

In the case of CN and C$_2$N, DRX shows the presence of the characteristic peak associated with interplane spacing^46,52,56,80^. However, this peak exhibits some specific features. First, it only appears after washing the samples. Second it is quite broadened. As TEM demonstrates the very high quality of the flakes, we consider that the broadness of the peak is likely to be due to a broad distribution of interplane spacings. This originates from the fact that the layers grow individually, getting stacked during the washing steps. This may induce an irregular spacing after drying. This may also ease the inclusion of metallic cations inside the coordinating holes. Indeed, a few percents of remaining K and Ca (a commonly observed impurity in commercial-grade potassium) are present in our CN and C$_2$N samples. The fact that our materials are directly obtained as single/few layers ones confirms the occurrence of very efficient mechanisms limiting its extension along the *z* axis (Fig. 1c). This confirms the proposed double confinement effect by (i) the surface of the potassium and (ii) the insoluble metal salts on top.

The first example of COF we describe (CN carbon nitride) has already been synthesized using a related protocol (reduction of cyanuric chloride with sodium under high pressure conditions in the presence of nickel as a catalyst^32–34^. However, the morphology of the material we obtained appears as very different. In our case, very large, well defined and seemingly defect-free 2D layers are obtained. These differences might be related to the difference in reactions conditions, and/or to the smaller ionic radii of Na$^+$ (Na being the reducing agent used in the previously cited studies) vs. K$^+$. Interestingly, our process led to a material that easily redisperses in water.

The same features were also observed for all three COFs described in the present work, i.e. very large, and seemingly defect-free flakes with good composition analyses. Accordingly, a comparison between the EDS and XPS analyses of CN, C$_2$N, and CTF shows a continuous decrease of the relative amount of nitrogen from CN to CTF, in accordance with the corresponding respective theoretical nitrogen contents in their structures.

For the first time, the flakes described in our work are sufficiently large and defect-free to behave as single-crystals, as evidenced by polarized light microscopy analyses.

TEM confirms the exceptional quality/crystallinity of the 2D layers of all three COFs. The best dispersions (thinnest and largest flakes) were observed upon treatment of the as prepared materials with HCl, in comparison with the other dispersant that we used. Aqueous HCl is thus the best choice for the suspension/processing of these materials, in line with previous observations^67,68^. This may be due to partial protonation of embedded nitrogen atoms, inducing electrostatic repulsions between flakes. This is attested by the observation of signals that could be attributed to (N–H)$^+$ bonds on the infrared spectra of CN and C$_2$N. This may also explain the presence of higher binding energy components in the N 1s/C 1s XPS analyses. Interestingly, thin flakes of the three COFs that we synthesized exhibit different intrinsic colors, brown for CN, greenish-brown for CTF, whereas C$_2$N exhibits a deep orange one. This evidences the smallest bandgap of the later.

It should be underlined here that for all the COFs that we synthesized, the whole materials are made of flakes such as the ones shown on the different figures (both in the paper and in the SI). Indeed, all the observed areas on several TEM grids from different syntheses do show similar objects. Improving the stability of the metallic interface by using potassium-mercury amalgam considerably improves the quality of the C$_2$N flakes, as expected for a surface-confined growth process. For the first time, millimeter-scale flakes of C$_2$N were obtained, an exceptional feature in the field of COFs. The successful synthesis of three different 2D networks, using very different building blocks shows that our metallic potassium-based process appears as quite general. This opens the door towards the synthesis of COFs with more complex structures.

## Conclusion

A simple protocol for the synthesis of graphene-related COFs was designed and exemplified on three different examples.

The exceptional quality of the resulting materials (in terms of flake sizes and defect-free nature) shows that the proposed *double confinement strategy* approach appears interesting for the synthesis of high-quality 2D materials. This high quality, along with the soft conditions used and common laboratory equipment makes our process a significant improvement in the field.

The fact that the characteristic X-ray diffraction peak around $26^{\circ }$ is only observed on washed samples tends to indicate that the growth of these 2D materials occurs as single/few layers, a new feature for wet syntheses of 2D materials.

These materials hold great promise in the field of nanoelectronics (high band gap support for graphene, transport studies, *etc*...). They are also interesting for photophysics, due to (i) the large size of the crystalline domains, (ii) their low defects content and (iii) their direct synthesis as highly exfoliated sheets. These studies are underway and will be reported in due course.

## Methods

2, 3, 5, 6-Tetrachloropyrazine was purchased from Chemieliva, and used as received. All other reagents were from TCI and used as received.

### Synthesis of CN

A chunk of metallic potassium (about 1 g) is weighted in a closed vial under argon. 20 mL of anhydrous benzene or toluene are then added, still under argon. A slight excess (vs. potassium) of cyanuric chloride (about 1.7 g) is then added, and the media is brought to reflux for 24 h under argon. Shortly after the beginning of the synthesis, the initially shiny potassium surface rapidly gets wrapped by a light brown deposit. During the course of the reaction, this solid material expands considerably, the flask being nearly completely filled with a brown solid at the end of the synthesis.

The material if filtered, and the solid is washed with acetone. These organic fractions are combined and evaporated. The resulting dark brown, oily material is weighted to estimate the yield of the reaction.

The complementary solid is then suspended in water, and filtered. This operation is repeated one more time. CN is obtained as a light brown solid.

### Synthesis of C_2_N

A chunk of metallic potassium (about 1 g) is weighted in a closed vial under argon. 20 mL of anhydrous benzene or toluene are then added, still under argon. A slight excess (vs. potassium) of tetrachloropyrazine (about 1.5 g) is then added, and the media is brought to reflux for 24 h under argon. Shortly after the beginning of the synthesis, the initially shiny potassium surface rapidly gets wrapped by a solid black deposit. During the course of the reaction, the solid material considerably expands (compared with the initial size of the potassium chunk, the flask being nearly completely filled with a black solid at the end of the synthesis.

The supernatant is filtered off, and the black solid is then washed with acetone. The organic solvent fractions are then combined and evaporated. The resulting dark brown oil is then weighted to assess the yield of the reaction.

The complementary black solid is then washed twice with deionized water, to remove soluble by-products (such as KCl).

### Synthesis of CTF 2D material

A chunk of metallic potassium (1 g) is weighted in a closed vial under argon. 60 mL of anhydrous benzene or toluene are then added, still under argon. Tris(bromophenyl)triazine (3.38 g) is then added, and the media is brought to reflux under argon for 3 days. Note that the media remains as a suspension, as the starting triazine product is poorly soluble. The initially shiny potassium surface rapidly gets purple, and then covers with a light brown solid. As the reaction goes by, the solid material considerably expands.

At the end of the refluxing period, remaining metallic potassium is quenched by slow addition of ethanol under argon.

The solid is filtered, washed with dichloromethane, acetone, and water. The precipitate is then suspended in o-dichlorobenzene, and centrifuged (10,000 RPM, 3 min). The precipitate (mostly unreacted starting product) is discarded. The supernatant is then filtered, leaving CTF-2D material as light greenish-brown powder.

### Synthesis of C$_2$N network using mercury/potassium amalgam

To a 10 mL Schlenk tube flushed with argon, are added 0.345 g of metallic potassium and 4 mL of mercury at ambient temperature. The dissolution of potassium in mercury is complete within 30 min. The white solids that appeared during the process at the surface of the amalgam are wiped out, leaving a clean, shiny surface. A solution of 0.49 g of tetrachloropyrazine in 5 mL of anhydrous toluene is then added. The reaction media is heated at 110 $^{\circ }$C under argon for 7 days, resulting in the formation of a brown deposit on the surface of the amalgam . After cooling the system down to ambient temperature, the liquid amalgam is removed (using a pipette), and the remaining brown powder is washed with ethanol and water under argon.

C$_2$N is obtained as a brown-orange powder.

### Supplementary Information

Supplementary Information.

## Data Availability

The datasets used and/or analyzed during the current study are available from the corresponding author upon reasonable request.
