# Supplementary material for: A simple and efficient process for the synthesis of 2D carbon nitrides and related materials
Source: Sci Rep. 2023 Sep 18;13:15423. doi: 10.1038/s41598-023-39899-5 (PMC10507022; doi:10.1038/s41598-023-39899-5)
Supplement: Supplementary file 1 — Supplementary Information. [file 41598_2023_39899_MOESM1_ESM.pdf]

Supplementary Information

A simple and efficient process for the synthesis of 2D carbon nitrides and related materials

Cora Moreira Da Silva[1], Maxime Vallet[2], Clément Semion[3], Thomas Blin[1], Romuald Saint-Martin[1], Jocelyne Leroy[4], Diana Dragoé[1], François Brisset[1], Cynthia Gilet[5], Régis Guillot[1], and Vincent Huc[1,*]

[1]Université Paris-Saclay, CNRS, Institut de Chimie Moléculaire et des Matériaux d'Orsay,91405, Orsay, France
[2]Université Paris-Saclay, Ecole Centrale Sup'Elec, France
[3]Université Paris-Saclay, ONERA, CNRS, Laboratoire d'Etude des Microstructures, Châtillon, 92322, France
[4]Université Paris-Saclay, CEA, CNRS, NIMBE, LICSEN, 91191, Gif-sur-Yvette, France
[5]CNRS-Institut de Biologie Intégrative de la Cellule (I2BC), France
[*]vincent.huc@university-paris-saclay.fr

General:

2, 3, 5, 6, tetrachloropyrazine was purchased from Chemieliva, and used as received. All other reagents were from TCI and used as received.

Toluene was dried over CaH_2.

XPS results were acquired using a Kratos Axis Ultra DLD spectrometer with a monochromatic Al Kα (1486.6 eV) X-ray source and a charge compensation system. Spectra were collected using a pass energy of 160 eV for survey and 40 eV for core levels.

TEM samples were prepared by dropping one or two droplets of a water (or HCl/EtOH) suspension of the flakes onto the TEM grids previously deposited on absorbing paper, to rapidly flow out excess solution.

Most TEM studies were performed using a FEI-CM 20 microscope, operating at 200 kV.

The sample shown on Figure 6c was studied by transmission electron microscopy (TEM) using a JEOL JEM-1400 microscope operating at 120 kV. The samples were stained using 2 % uranyl acetate and deposited onto copper grids covered with formvar film (400 mesh). The excess liquid was blotted off using filter paper, and the grids were dried before observation.

Images were acquired using a post-column high-resolution (9 megapixels) high-speed camera (RIO9; Gatan) and processed with Digital Micrograph (Gatan) and ImageJ (open-source software, Research Services Branch, National Institute of Mental Health, Bethesda, MD).

The experiments Figures 6d-f, 9d-g, 10d-f, S2-5, S2-8 and S3-6 were realized using a TITAN3 G2 TEM within the MATMECA consortium at Centrale Sup'Elec, partially funded by the French ANR under contract number ANR-10-EQPX-37

S1: Additional characterizations of CN

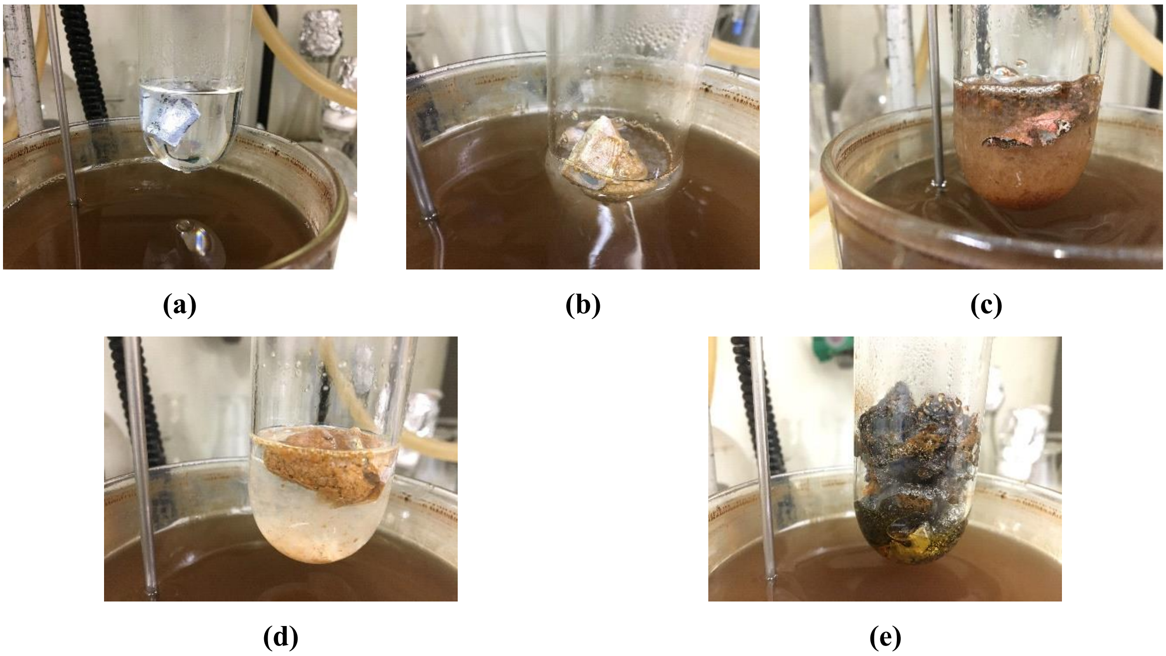

Fig. S1-1: Evolution of the reaction media during CN synthesis (solvent: benzene)

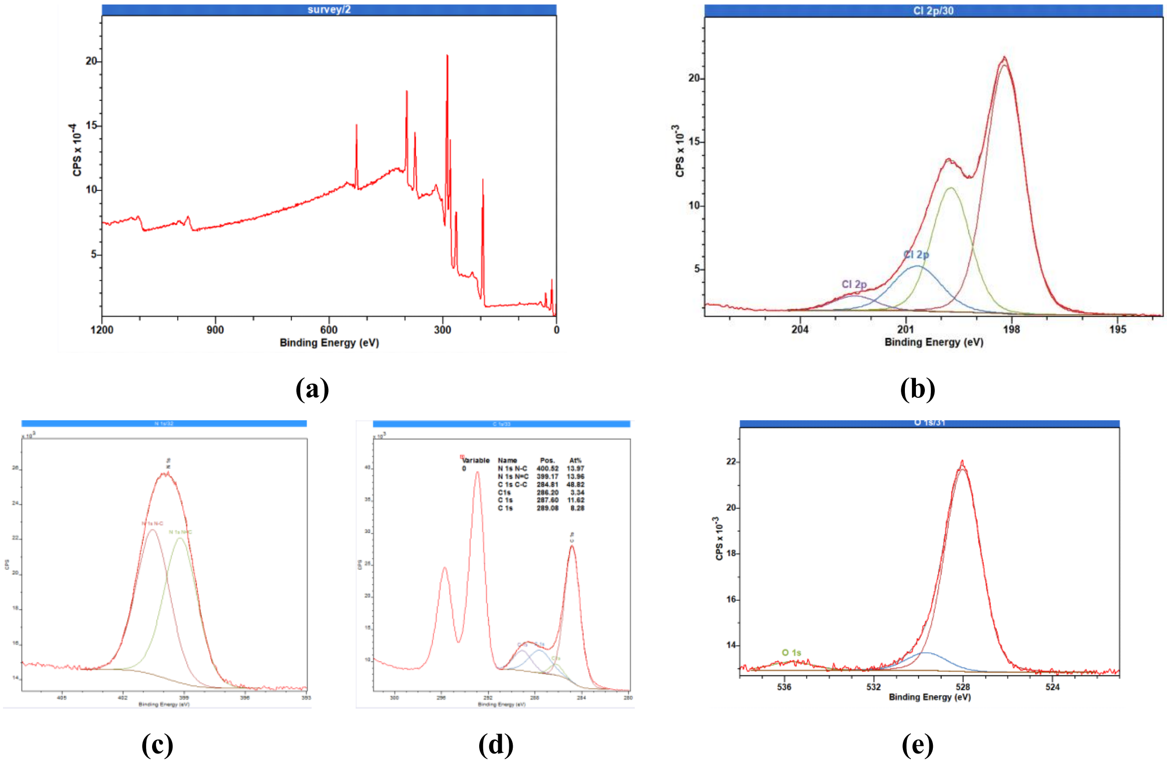

Fig. S1-2: XPS analysis of crude CN with signal of **(a)** survey, **(b)** chlorine, **(c)** nitrogen, **(d)** carbon and **(e)** oxygen

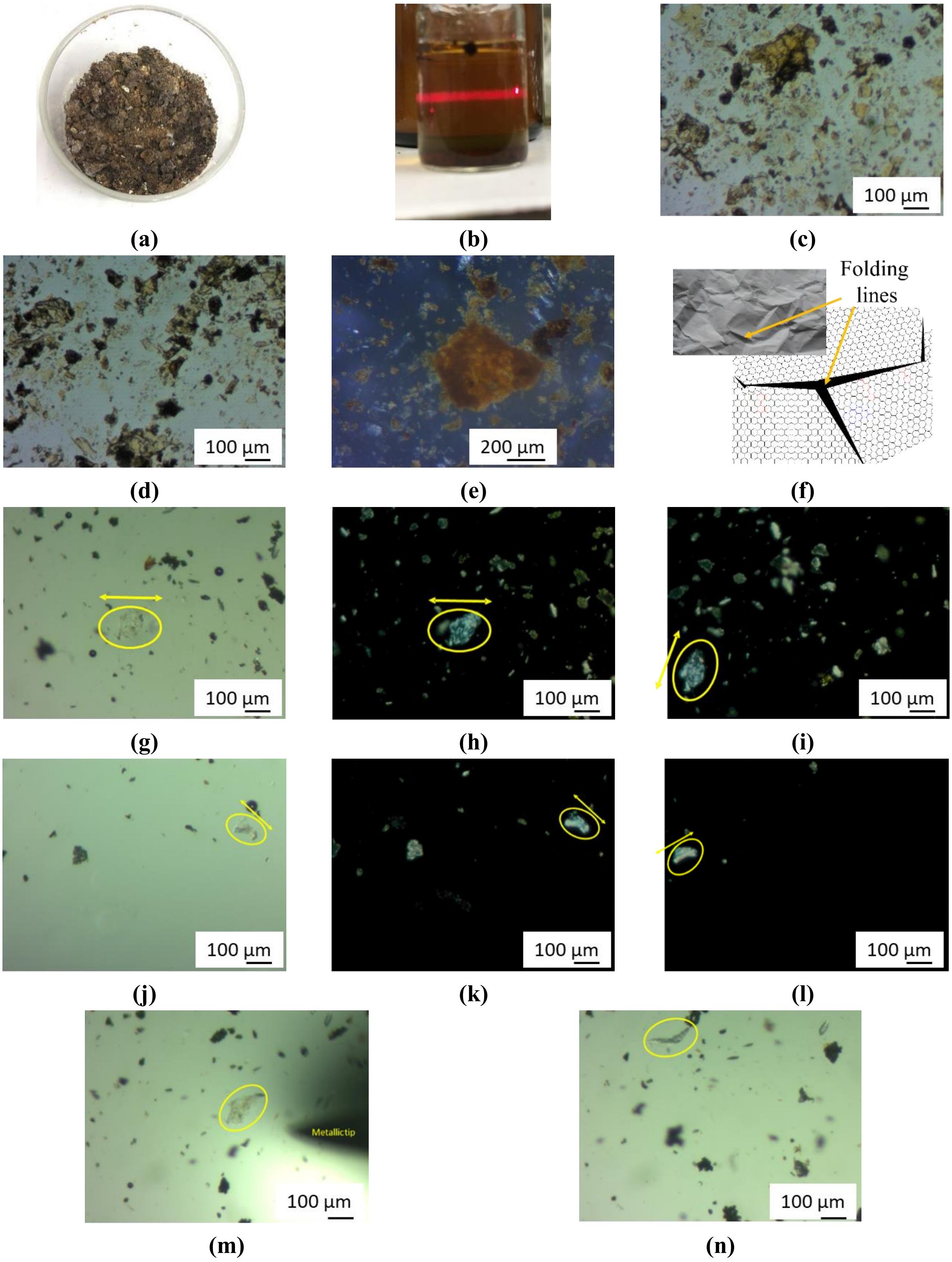

Fig. S1-3: **(a)** ~~The crude of~~ CN, **(b)** Water suspended CN showing the Tydall effect (*i.e.* phenomenon of scattering of incident light on particles of matter, of dimensions smaller than or comparable to the wavelengths of this light), **(c)-(e)** optical microscopy analysis showing large CN flasks. **(f)** A simple model showing how folds may give rise to different domains inside a single crystal flake.
Polarized optical microscopy analysis of CN flakes (yellow circles) at **(g)** and **(j)** normal light, **(h)-(l)** polarized light. **(m)-(n)** Handling experiment of a single flake. The highlighted flake (yellow circle) is pushed and tilted using a metallic tip. Note that this flake is the same than the one shown on Fig. 3b in the main text of the article.

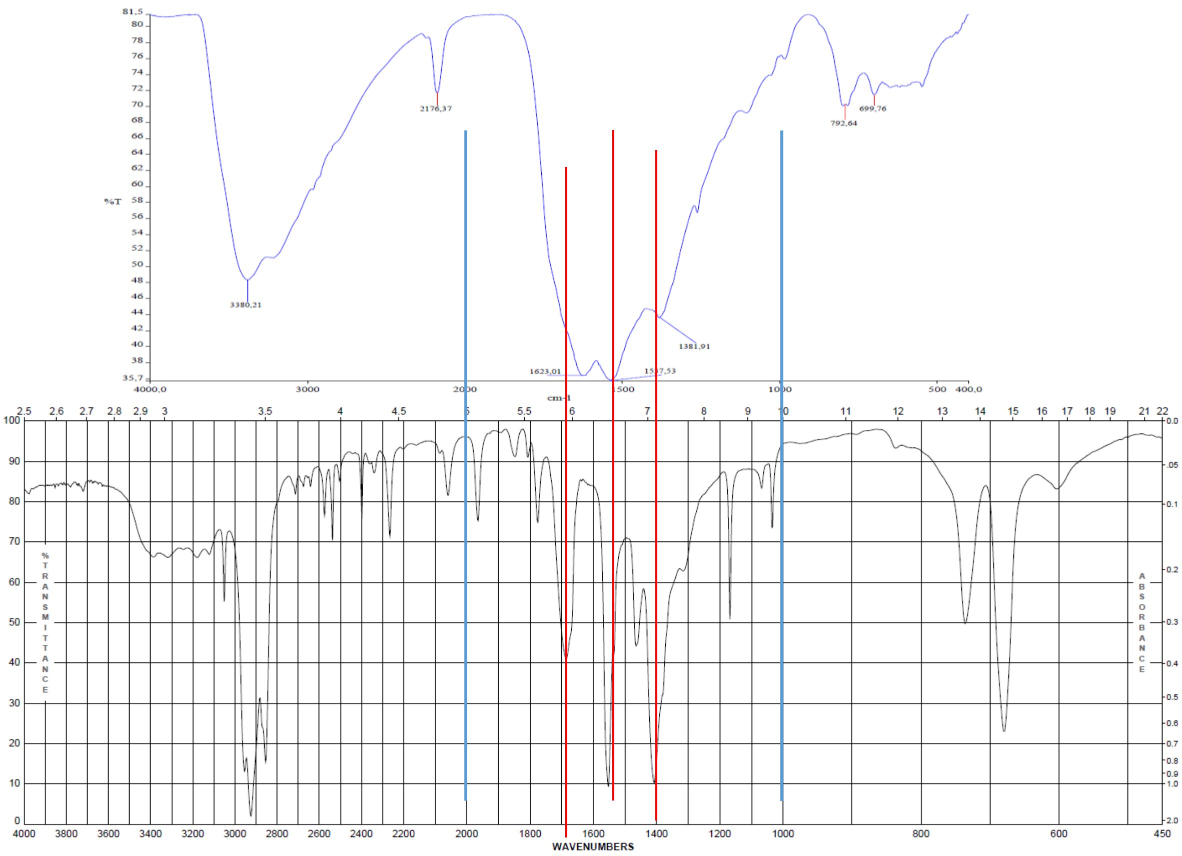

(a)

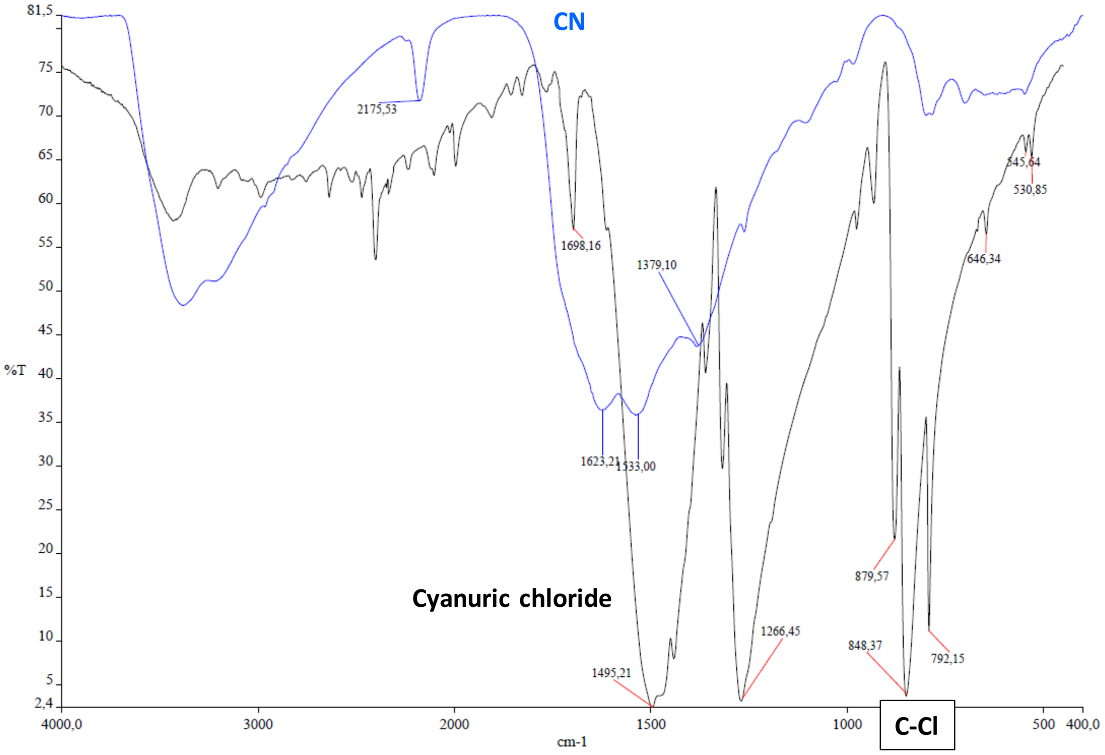

(b)

<u>Fig. S1-4:</u> Compared IR analysis of **(a)** CN (blue) and triazine (black) and **(b)** CN (blue) and starting cyanuric chloride (black). Blue lines (spectra alignment) and red ones (peaks comparition) are shown as a guide to the eye.

S1-5: Preparation/analysis of uranyl-loaded CN

To a suspension of 50mg of CN in 3 mL water was added 50mg of uranyl acetate, and the resulting yellow suspension was stirred at ambient temperature for three days.

An aliquot of this suspension was taken out and directly dropped on a Whatman paper, to remove excess water. The dried material was recovered with a spatula, further dried under vacuum and XPS-analyzed (referred as « CN-U »).

The remaining suspension was filtered, and extensively washed with water. The resulting washed solid was dried under vacuum and XPS-analyzed (referred as « CN-U washed »).

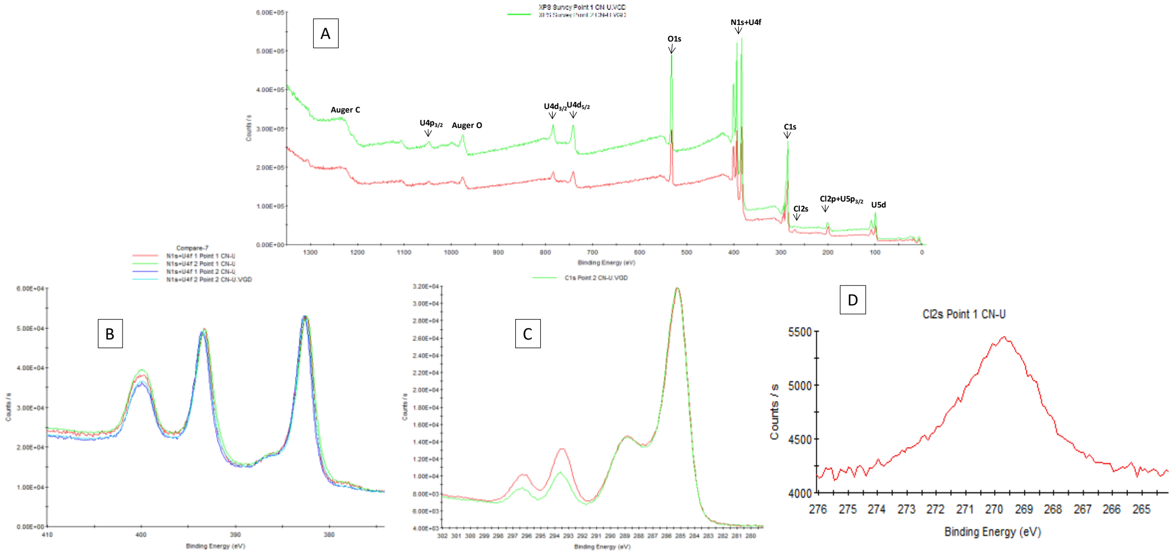

Fig. S1-5a: XPS analysis of CN-U. **(a)** Survey at two different locations of the sample, **(b)** N1s and U4f high resolution analysis and stability monitoring over time, **(c)** C 1s and K2p analysis, **(d)** Cl2P analysis.

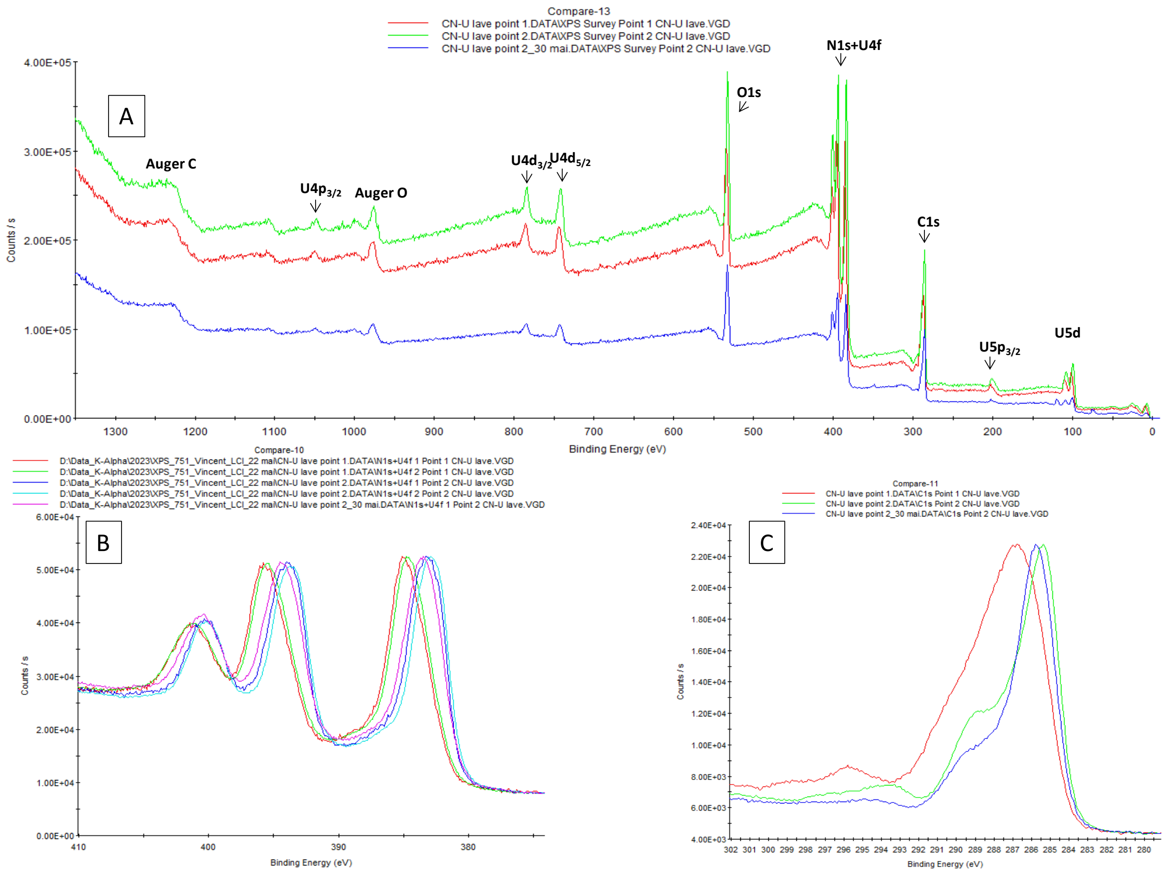

Fig. S1-5b: XPS analysis of CN-U-washed. **(a)** Survey at three different locations of the sample, **(b)** N1s and U4f high resolution analysis and stability monitoring over time, **(c)** C 1s and K2p analysis.

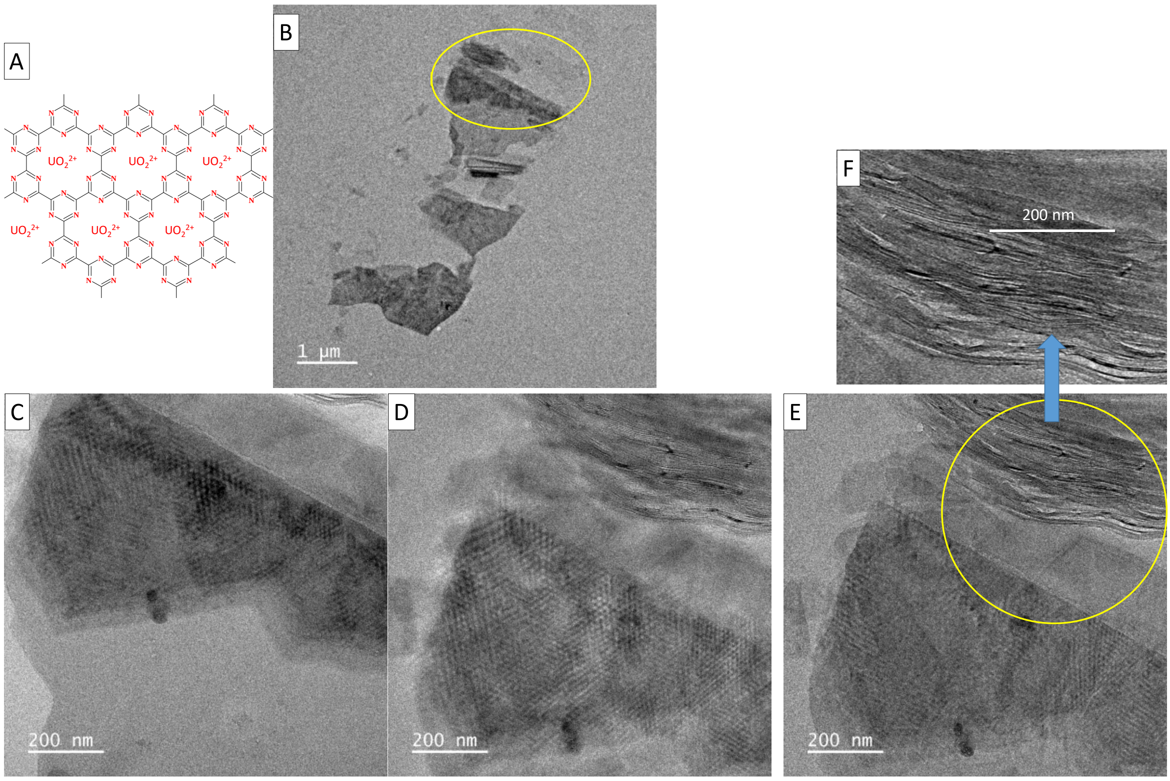

Fig. S1-5c: TEM analysis of a CN-U-washed flake. **(a)** Structure of the material, **(b)** the observed flake (analyzed area inside the yellow circle), **(c)-(e)** Moiré patterns, **(f)** zoom over the yellow circle highlighted area in (e), evidencing the layered structure of the material.

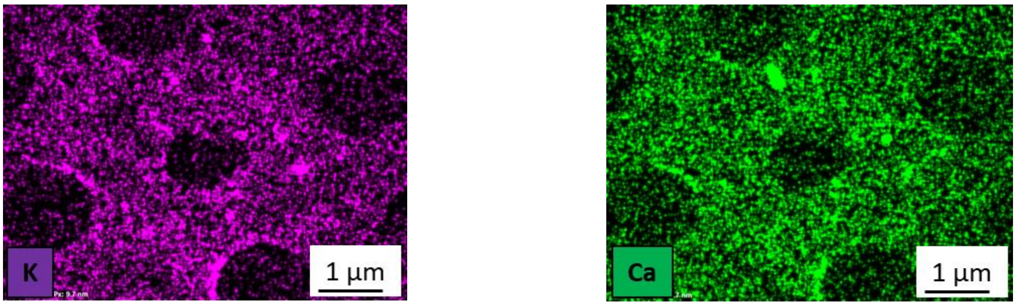

(a) (b)

Fig. S1-6: STEM-EDS mapping mode of **(a)** potassium and **(b)** calcium.

S2: Additional characterizations of C_2N

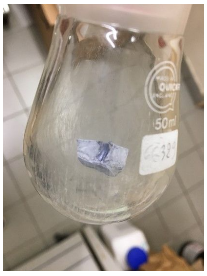

(a)

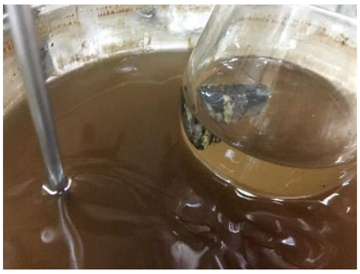

(b)

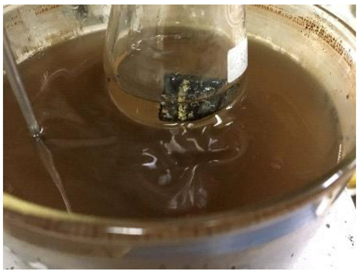

(c)

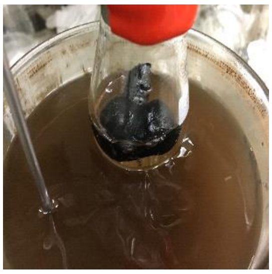

(d)

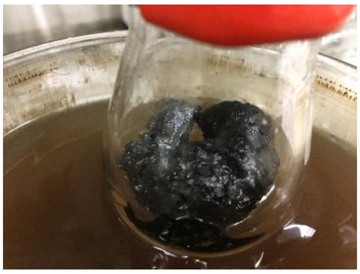

(e)

Fig. S2-1: Evolution of the reaction media during C_2N synthesis (solvent: benzene)

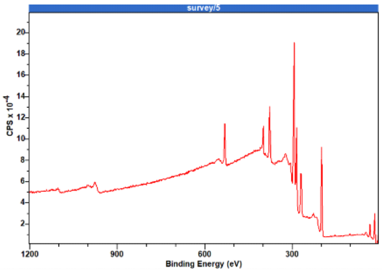

(a)

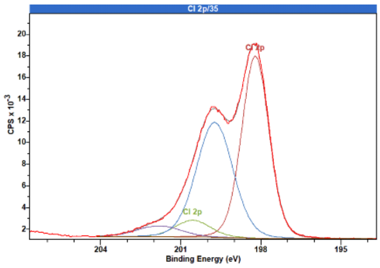

(b)

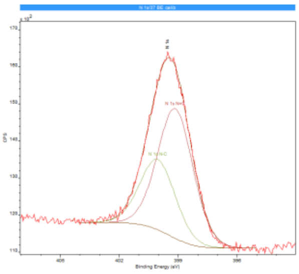

(c)

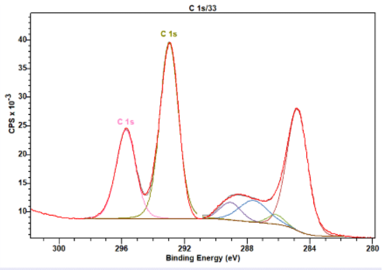

(d)

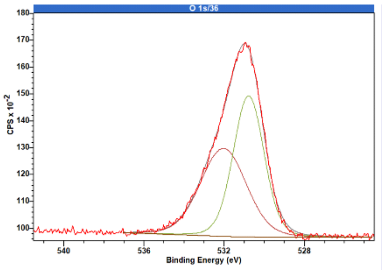

(e)

Fig. ~~S1-~~2: XPS analysis of crude CN with signal of **(a)** survey, **(b)** chlorine, **(c)** nitrogen, **(d)** carbon and **(e)** oxygen

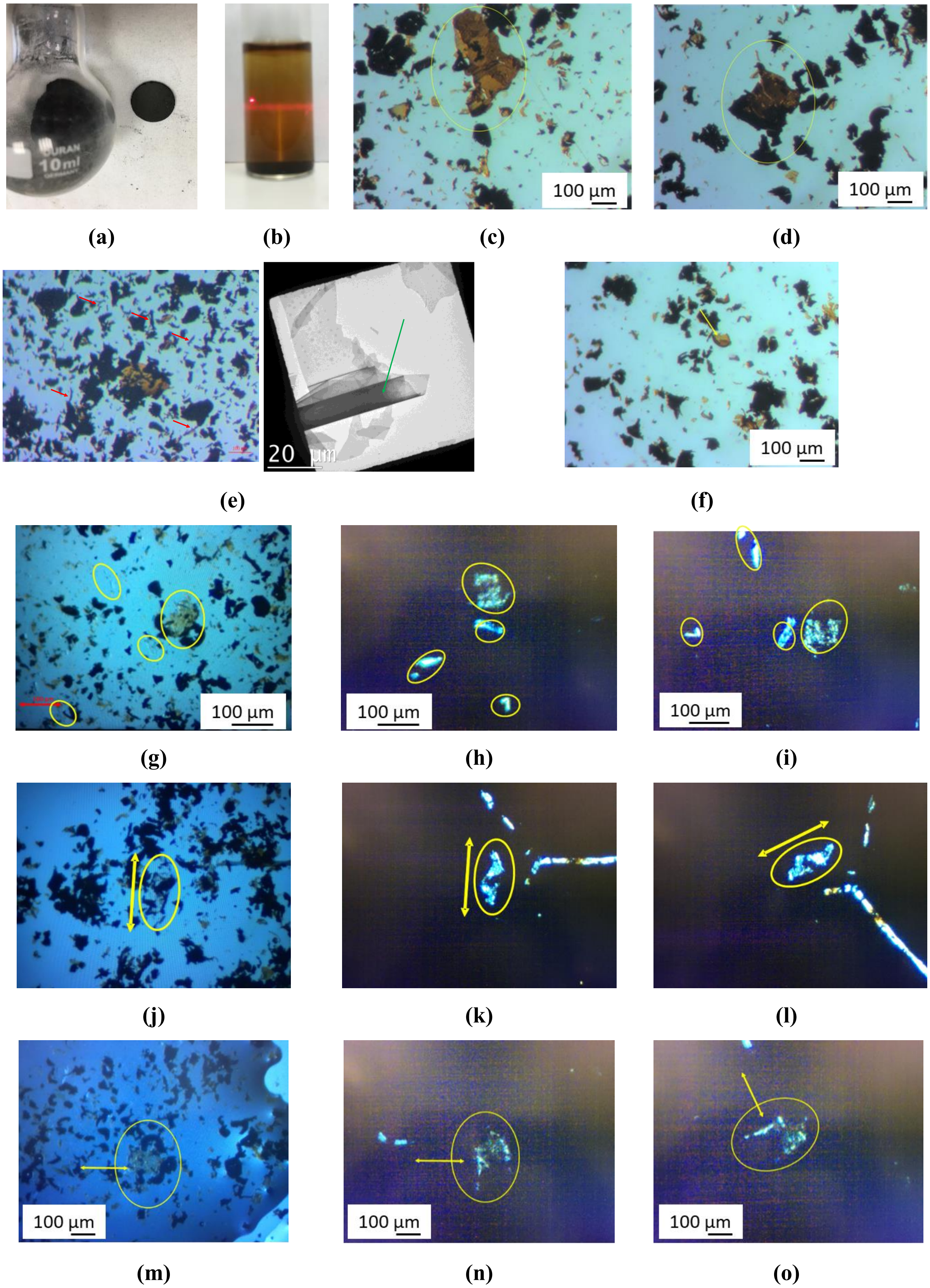

Fig. S2-3: **(a)** Washed C_2N, after the synthesis and ~~in~~ compressed pellet, **(b)** HCl suspended C_2N showing the Tydall effect, **(c)**-**(d)** compared optical microscopy appearance of two large and flat flakes (yellow circles) as a function of their thickness ((c) thin et (d) thicker). Optical observation of **(e)** tube-shaped objects (green/red arrows) observed by optical microscopy, along with the corresponding TEM micrograph) and **(d)** shell-shaped C_2N objects.

Polarized optical microscopy analysis of flakes (yellow circles) at **(g)**, **(j)** and **(m)** normal light, **(h)**-**(o)** polarized light.

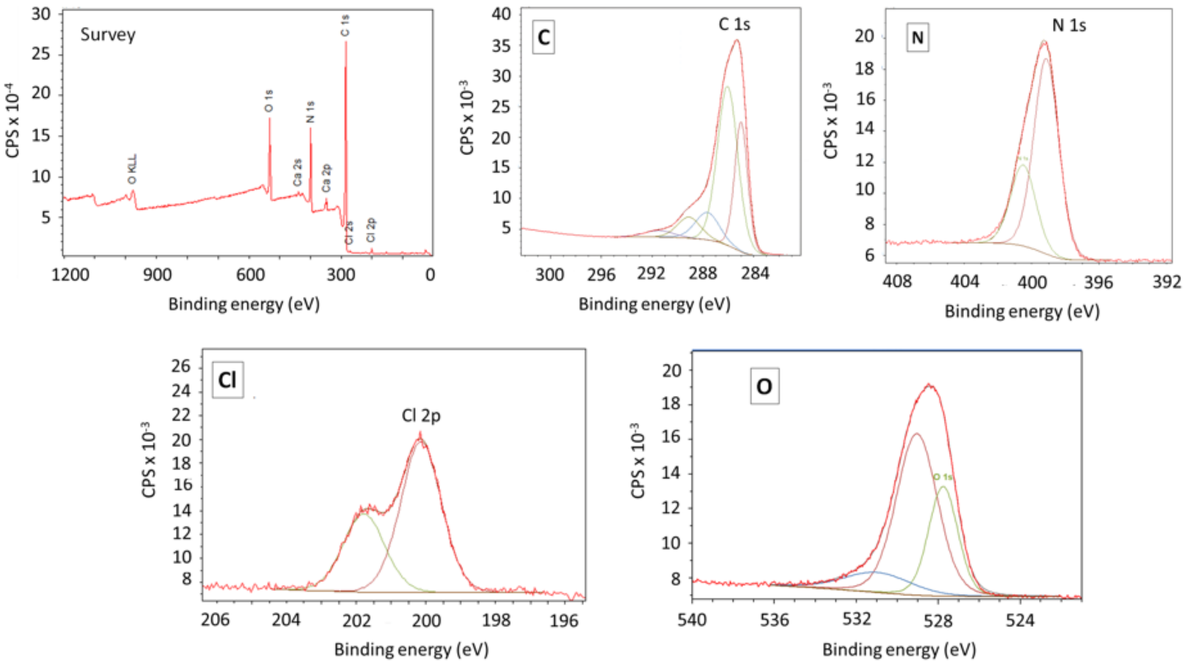

Fig. S2-4: XPS analysis of a washed C_2N sample with signal of **(a)** survey, **(b)** carbon, **(c)** nitrogen, **(d)** chlorine and **(e)** oxygen.

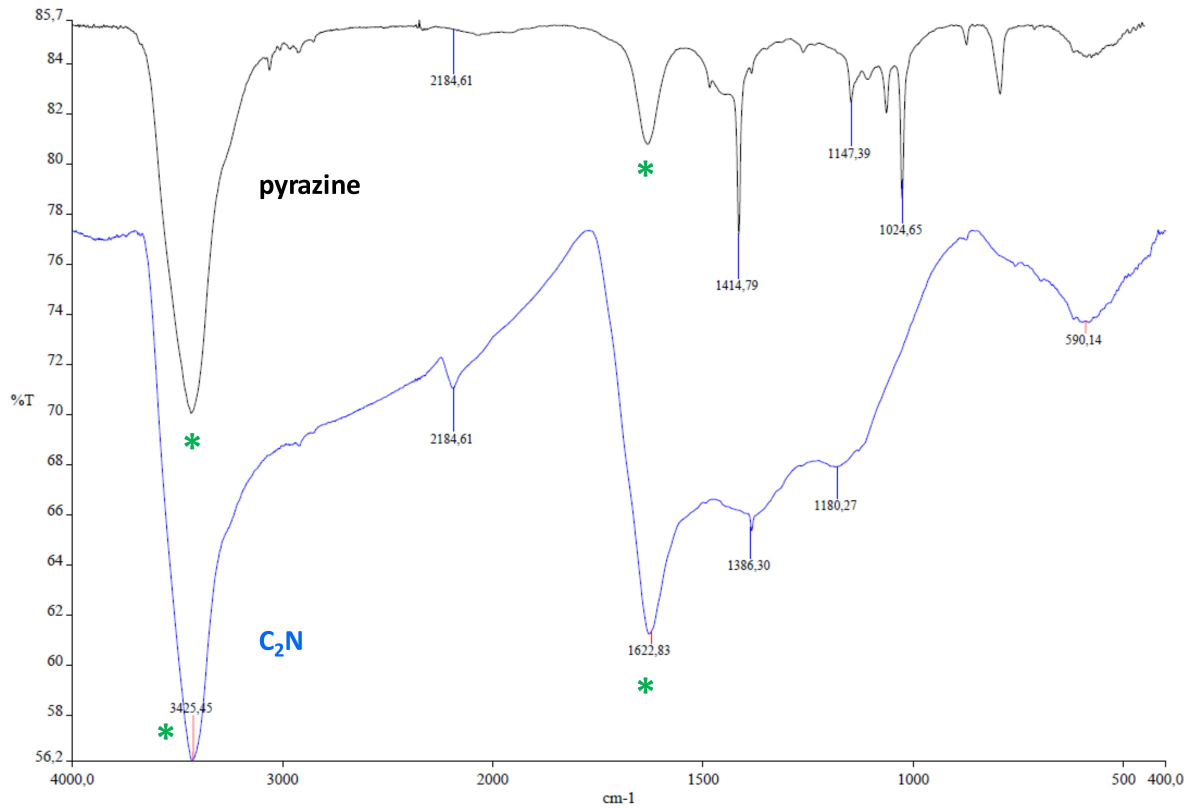

(a)

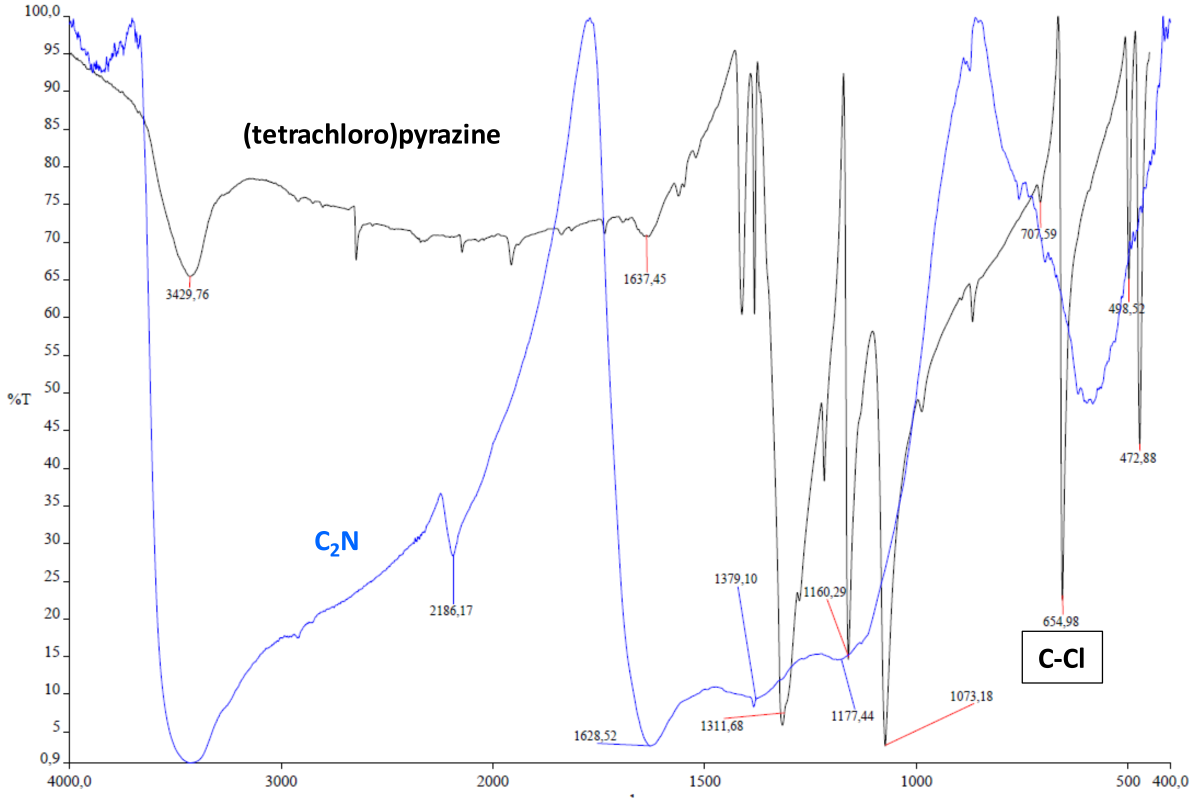

(b)

Fig. S2-5: Compared IR analysis of **(a)** pyrazine (black) and C_2N (blue) and **(b)** starting tetrachloropyrazine (black) and C_2N (blue). Peaks spotted with an asterisk correspond to water.

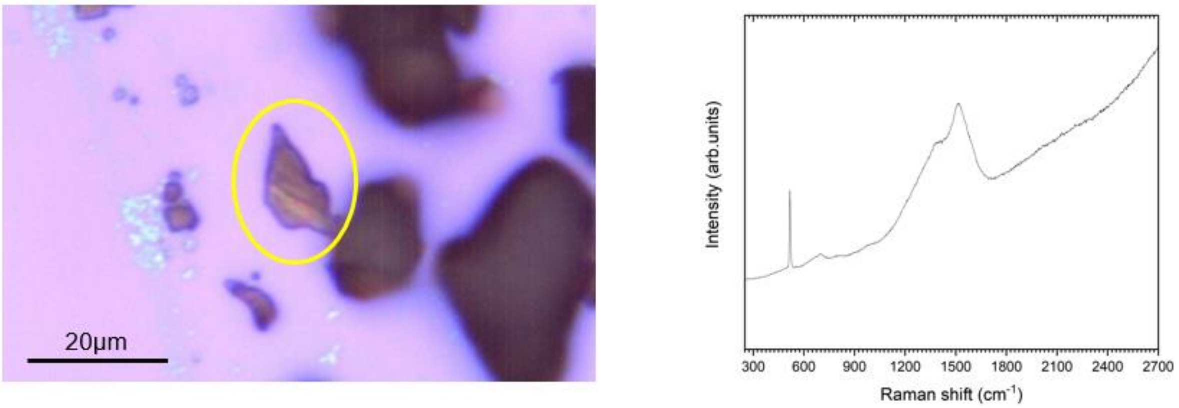

Fig. S2-6: Raman analysis of a single C_2N flake acquired during 15 s at λ_{laser}=532 nm – 50 mW – network: 1200t/mm

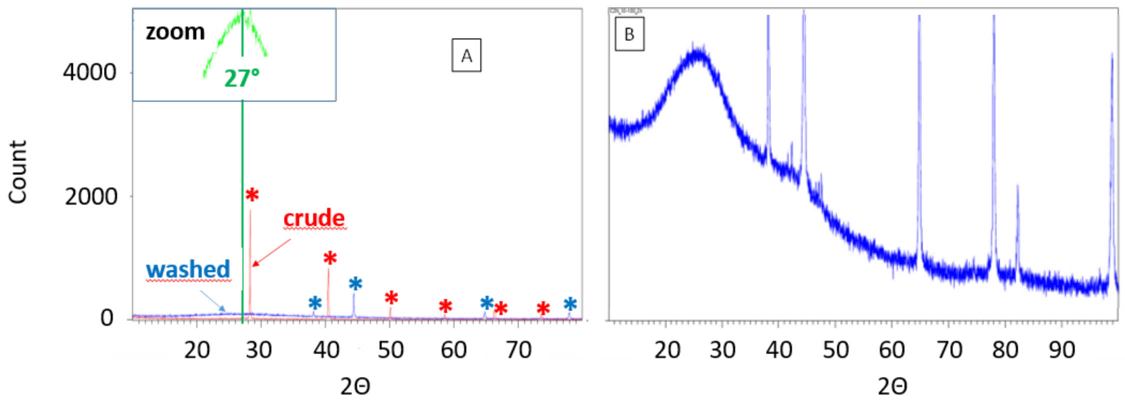

Fig. S2-7: **(a)** Compared powder XRD of a crude (red) and washed (blue) C_2N samples (same batch). In inset (green): zoom over the blue curve. Blue stars: signals from the Al sample holder, red stars: signals from KCl by products. **(b)** Powder XRD of the washed C_2N sample. Black stars: signals from the Al sample holder.

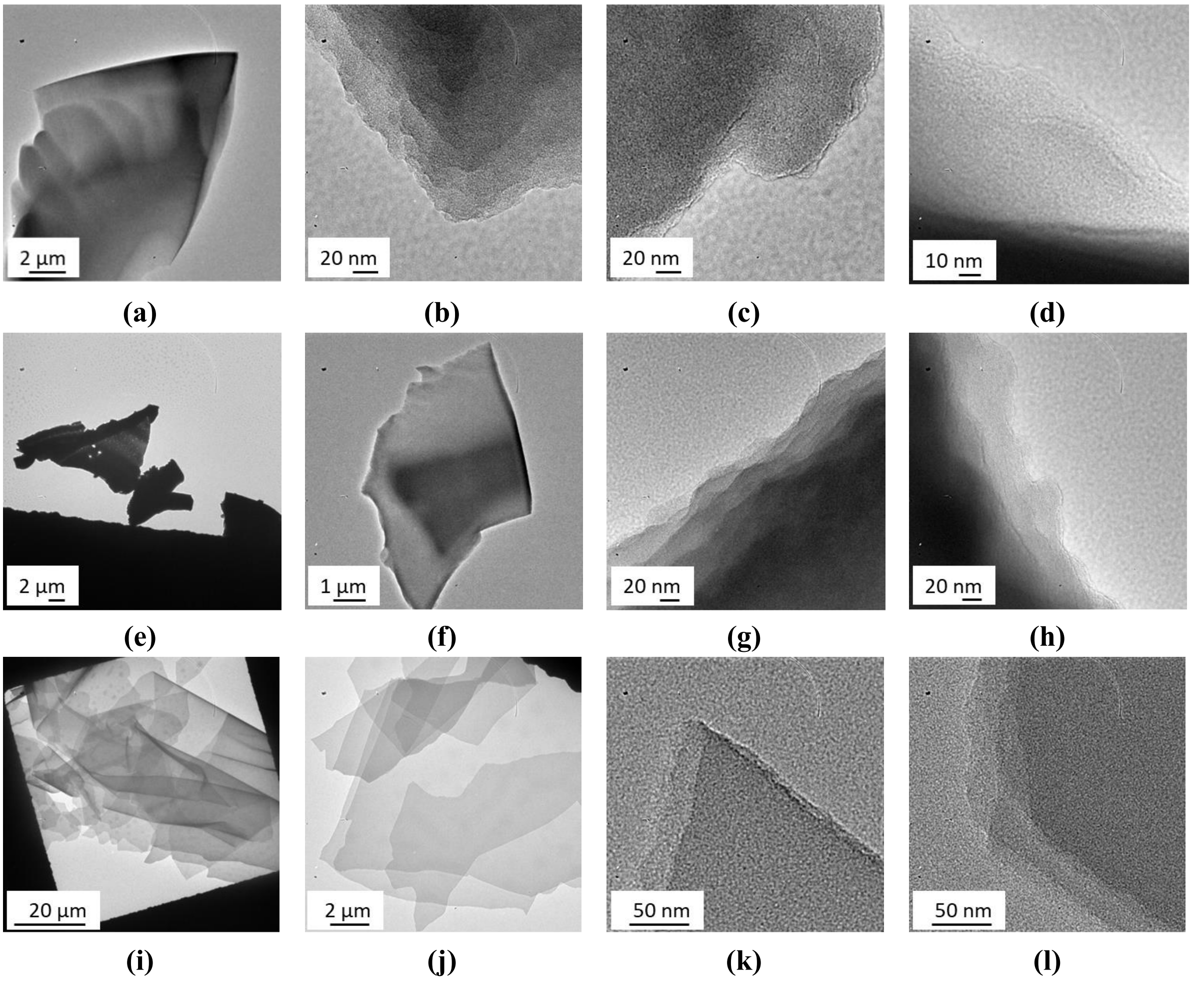

Fig. S2-8: TEM images of optimization of the solvents used for dispersing the C_2N carbon nitride, **(a)-(d)** in acetone: large, smooth flakes are observed. They appear as rather thin, being quite transparent to the e-beam. A detailed observation of the edges of these flakes shows that the material has a layered structure, in accordance with SEM observation. The layers appear as rather smooth, with few defects.
(e)-(h) in ethanol: presence of layered, large flakes of various thicknesses.
(i)-(l) in 1 M HCl.

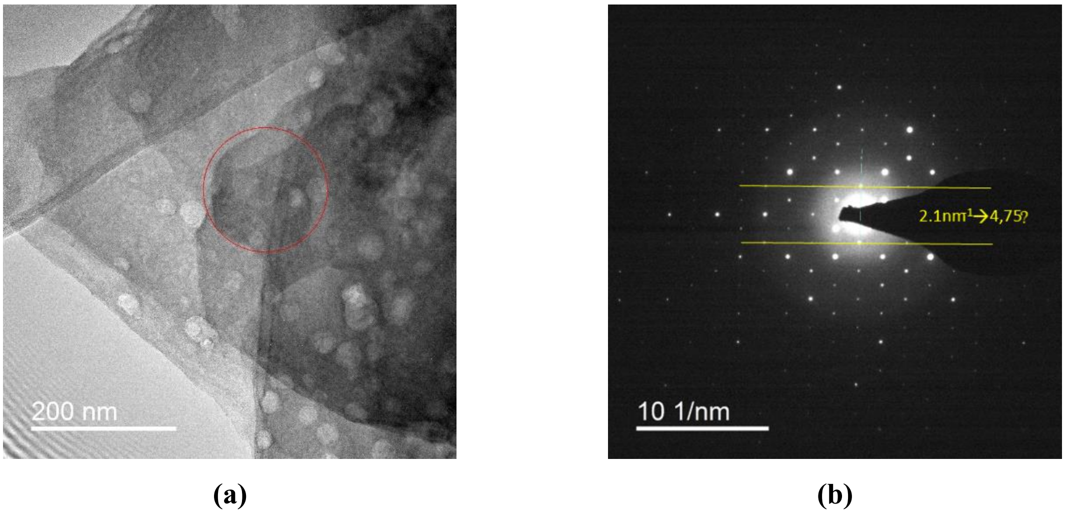

Fig. S2-9: Example of artifacts observed during C_2N carbon nitride SAED analysis. Holey TEM carbon grid used, suspended sample. **(a)** TEM image and **(b)** SAED acquired at the red circle in (a).

S2-10: preparation of uranyl-loaded C_2N.

To a suspension of 3mg of C_2N in 3mL of deionised water was added 5 mg of uranyl acetate.

The resulting suspension was stirred at ambient temperature overnight and centrifuged. The supernatant was separated, and the precipitate dried under vacuum. Uranyl-loaded C_2N is obtained as a black powder.

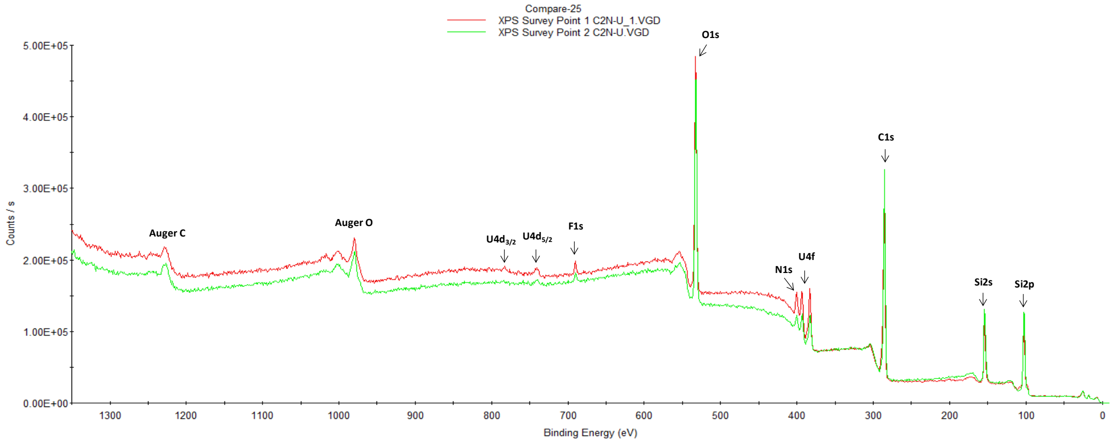

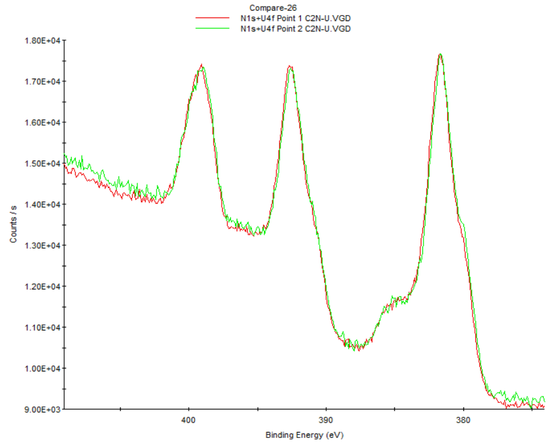

Point 1	Name	Peak BE	FWHM eV	Area (P) CPS.	Area (N)	Atomic %
	**N1s**	399,12	2,18	8242,84	69,01	**2,5**
	**U4f**	381,65	2,76	38142,06	11,65	**0,4**
	**C1s**	284,45	1,37	97210,45	1379,49	**50,1**
	**O1s**	532,07	1,33	116977,9	650,64	**23,6**
	**Si2s**	153,12	2,02	46105,99	643,79	**23,4**
**Point 2**	**Name**	**Peak BE**	**FWHM eV**	**Area (P) CPS.**	**Area (N)**	**Atomic %**
	**N1s**	399,01	2,11	5562,13	46,57	**1,6**
	**U4f**	381,58	2,83	26379,93	8,06	**0,3**
	**C1s**	284,44	1,35	104862,47	1488,08	**49,7**
	**O1s**	532,07	1,33	128778,3	716,27	**23,9**
	**Si2s**	153,13	2,02	52767,45	736,81	**24,6**

Figure S2-10: XPS analysis of an uranyl-loaded C_2N sample. For the two points analysed, the same N/U atomic ratio of 6 was found.

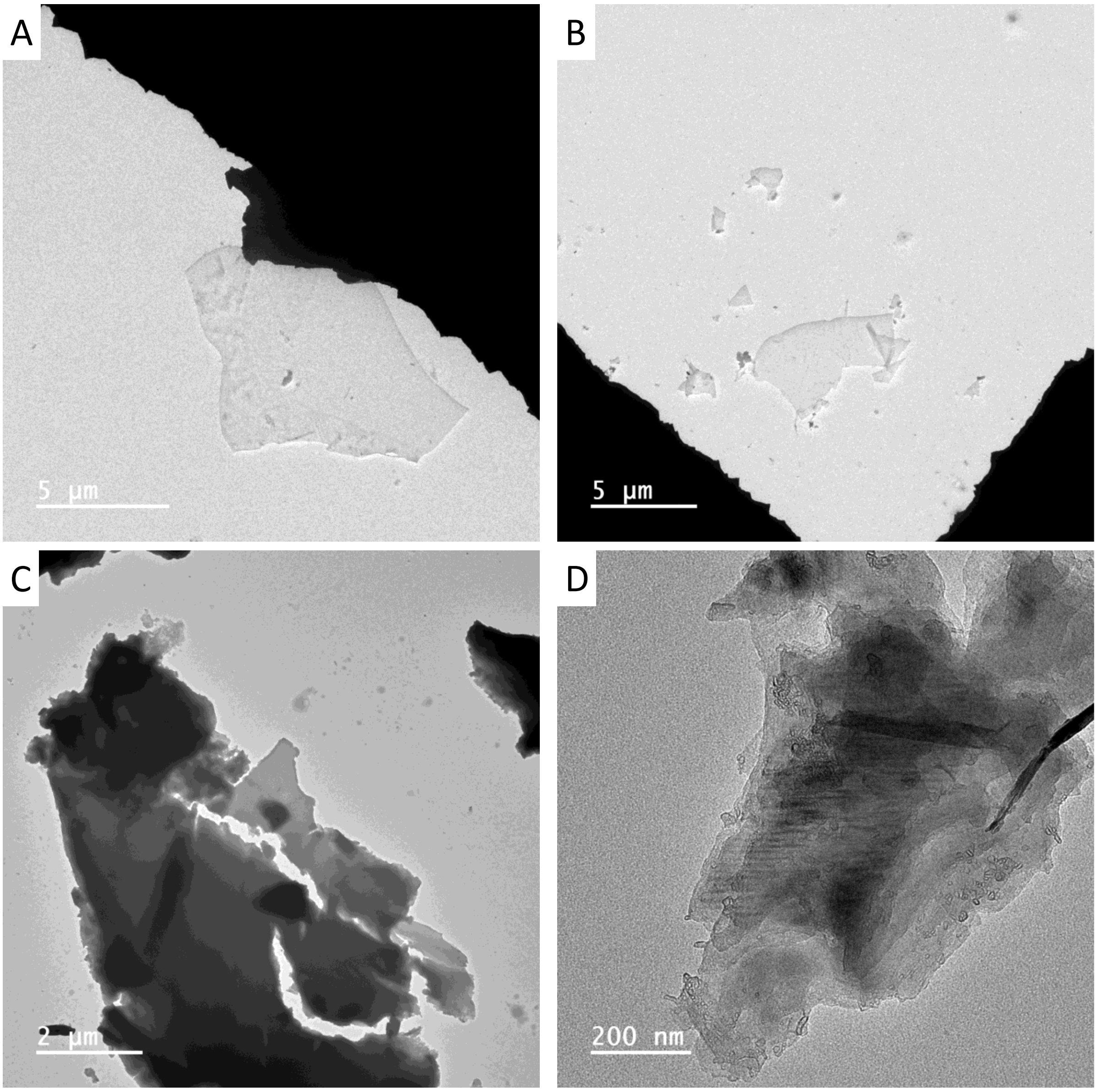

Figure S2-11: TEM analysis of an uranyl-loaded C_2N sample. Large, very contrasted flakes are observed. Flake D shows Moiré effects.

S3: Additional characterizations of CTF-2D material

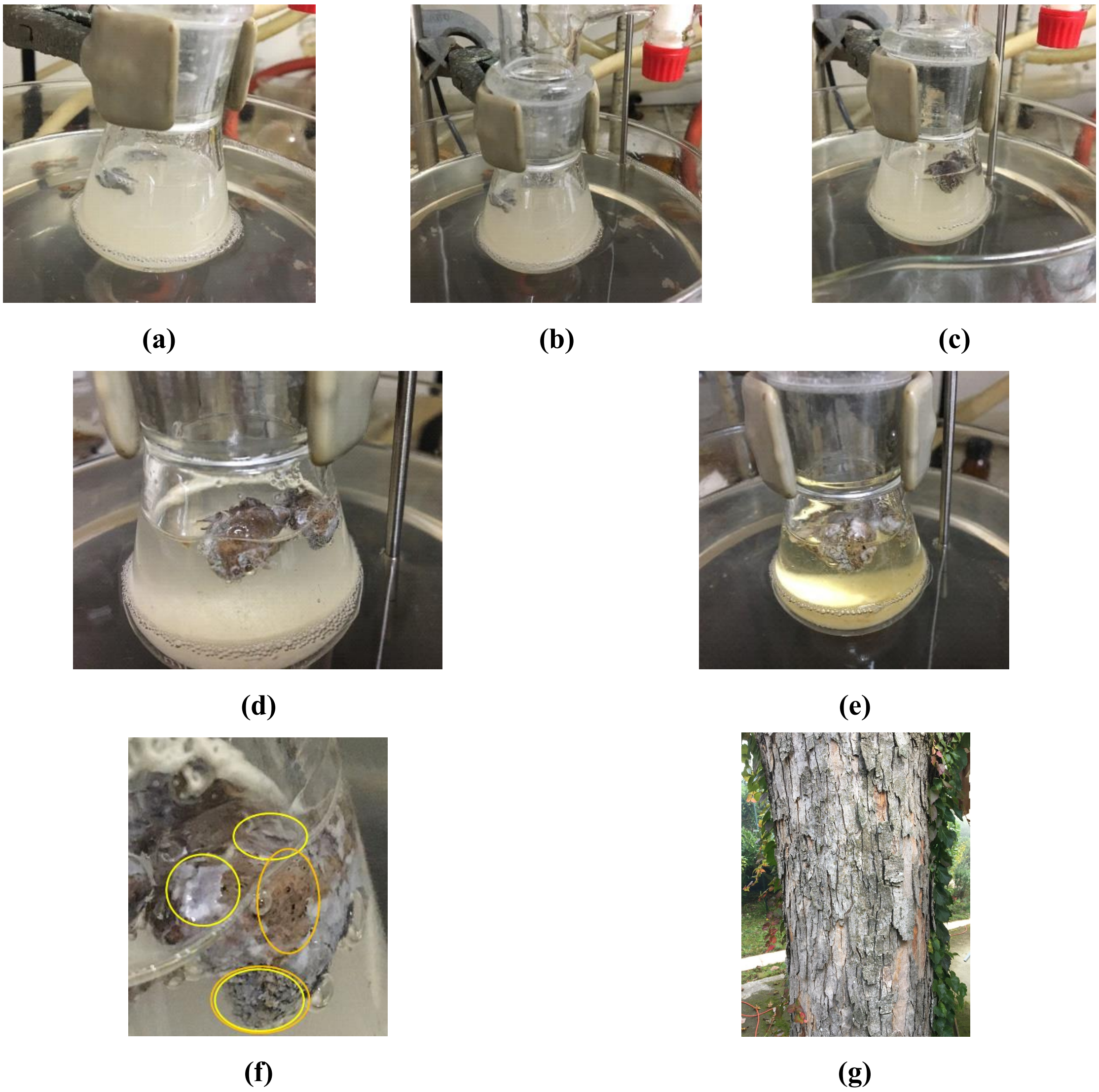

Fig. S3-1: **(a)-(e)** Evolution of the reaction media during the reaction of CTF-2D material. Bark-type growth of CTF organic network: **(f)** KBr (yellow circles) and 2D CTF (orange circles) scales, **(g)** analogy with bark.

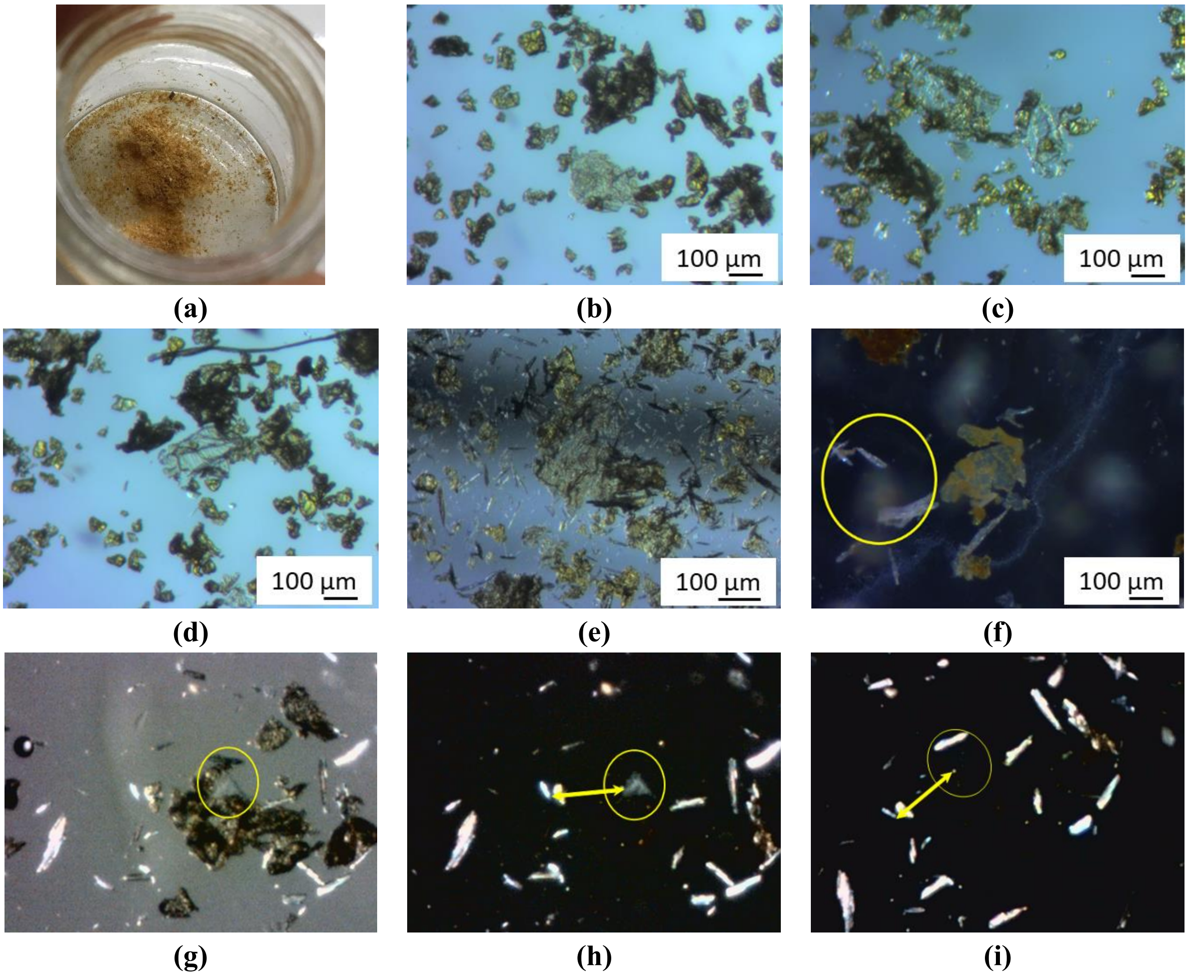

Fig. S3-2: CTF-2D material images of **(a)** ~~crude~~ and **(b)-(f)** ~~optical microscopy~~ (yellow circle: starting product).
Polarized light study of a thin TPTZ 2D flake (yellow circles) at **(g)** normal light and **(h)-(i)** polarized light.

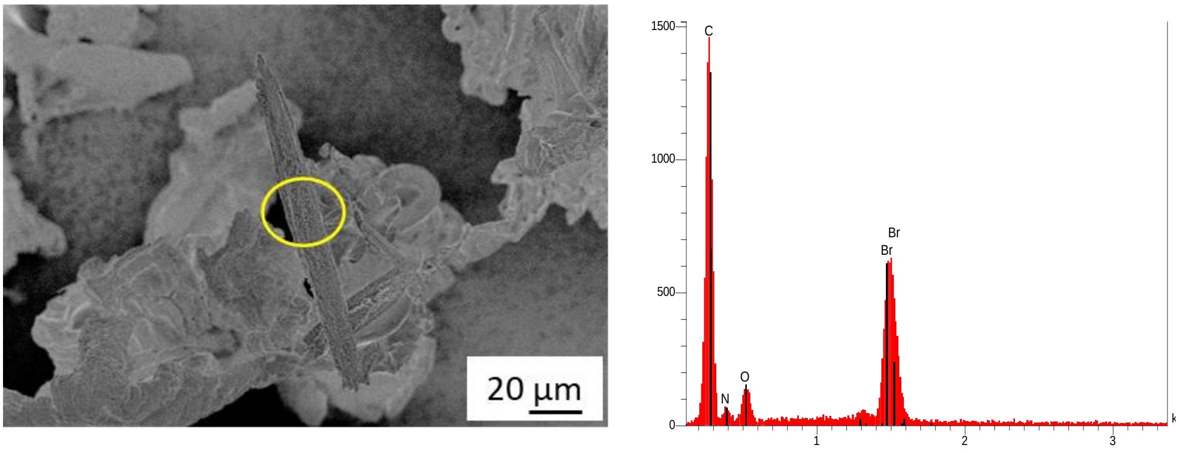

(a) **(b)**

Fig. S3-3: Analysis of a needle-shaped crystal of starting product **(a)** SEM image and **(b)** EDS analysis on yellow circle

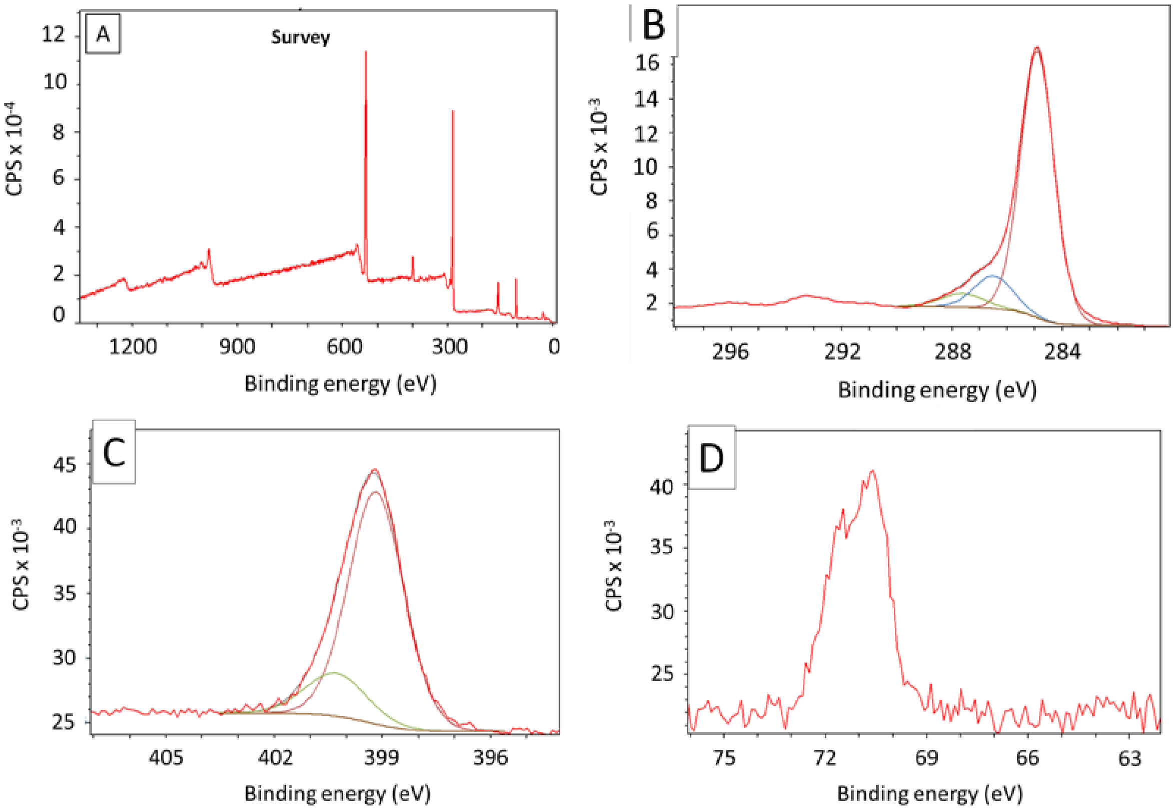

Fig. S3-4: XPS analysis of a washed CTF-2D material sample with signal of **(a)** survey, **(b)** carbon, **(c)** nitrogen and **(d)** bromine.

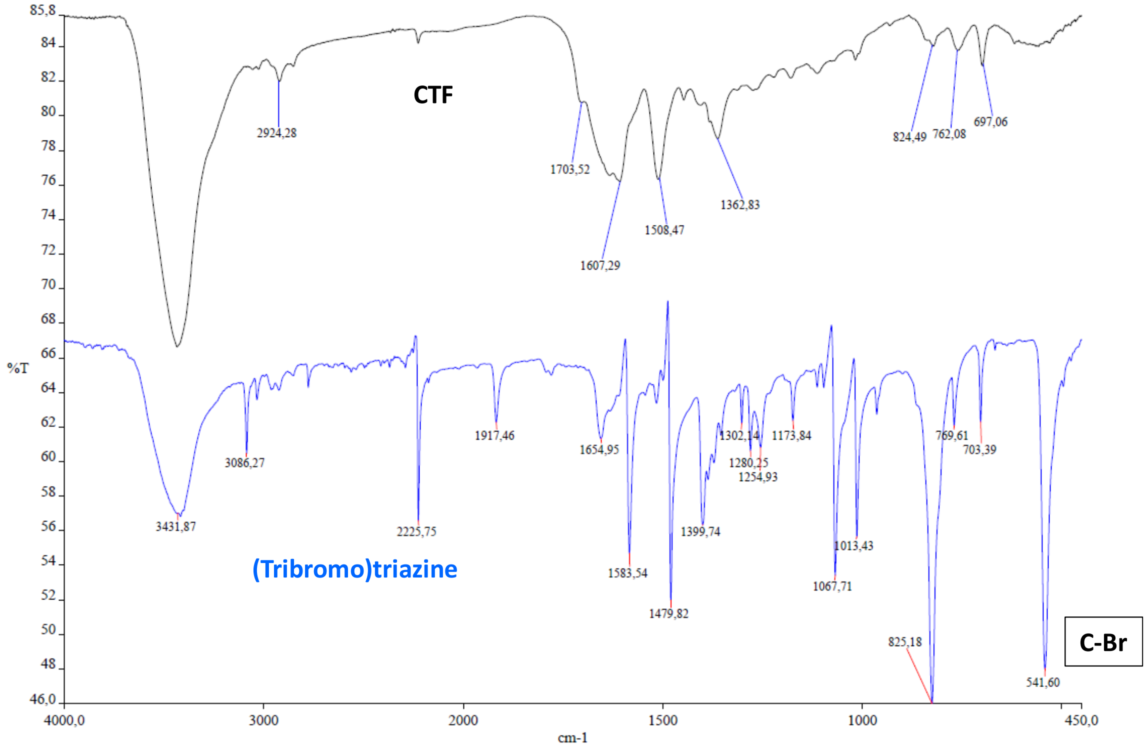

Fig. S3-5: Compared IR study of CTF (black) and starting (tris 4-bromophenyl)triazine (blue).

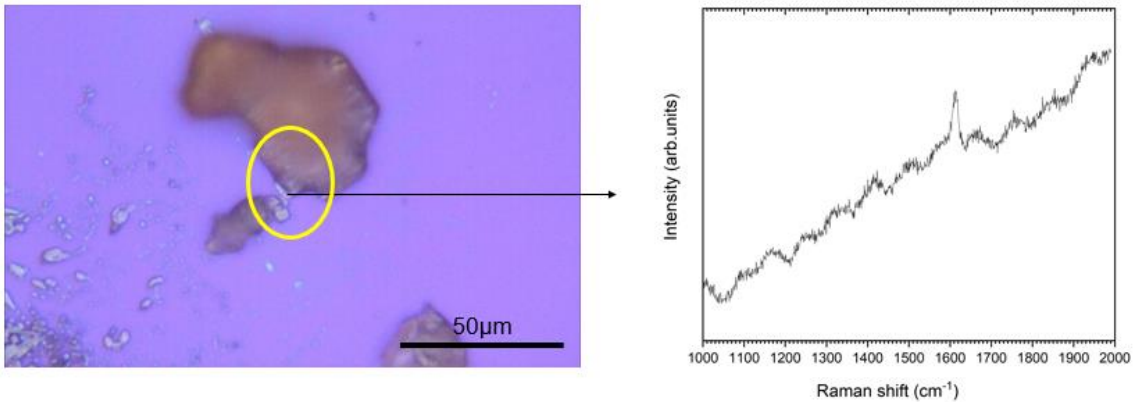

<u>Fig. S3-6:</u> Raman analysis of a single CTF flake acquired during 10 s at λ_{laser}= 785 nm – 25 mW – network: 1200t/mm

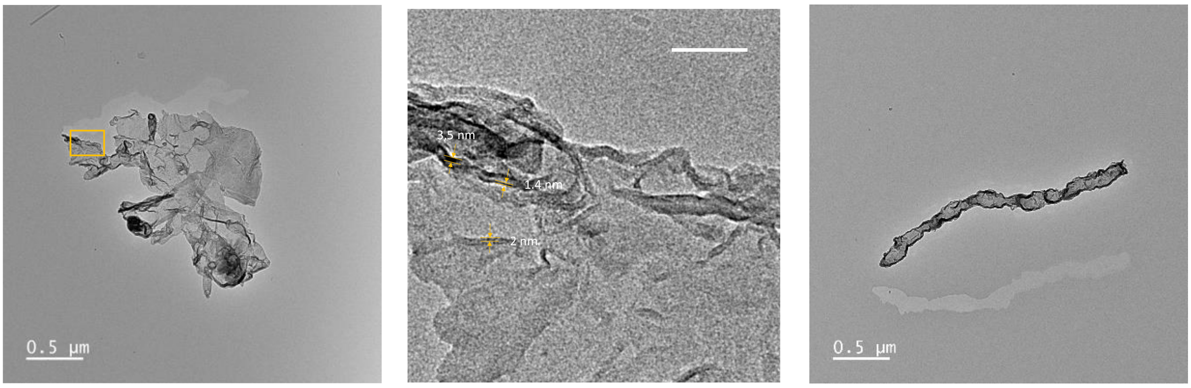

(a) **(b)** **(c)**

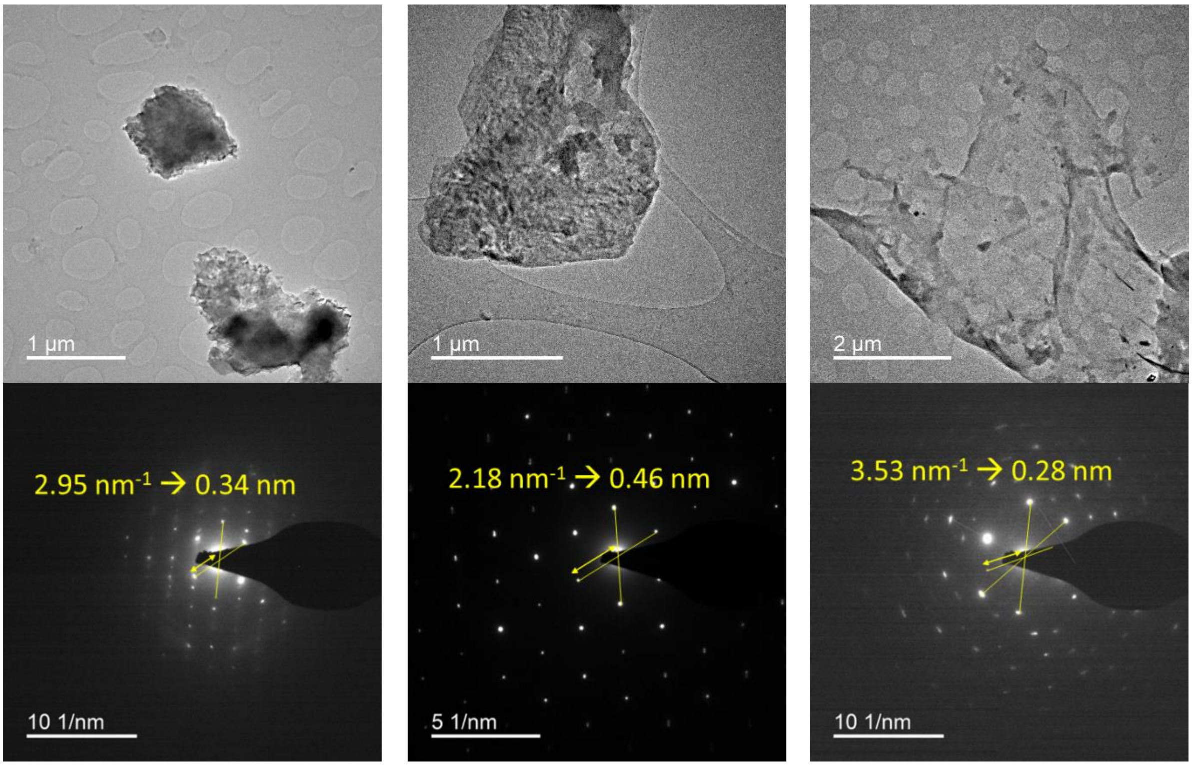

(d) **(e)** **(f)**

<u>Fig. S3-7:</u> TEM analysis of **(a)**-**(b)** highly folded CTF flake, **(c)** tube-shaped CTF flake and **(d)**-**(e)**-**(f)** artifacts during SAED analysis.

S4: Additional characterizations of C_2N using mercury/potassium amalgam

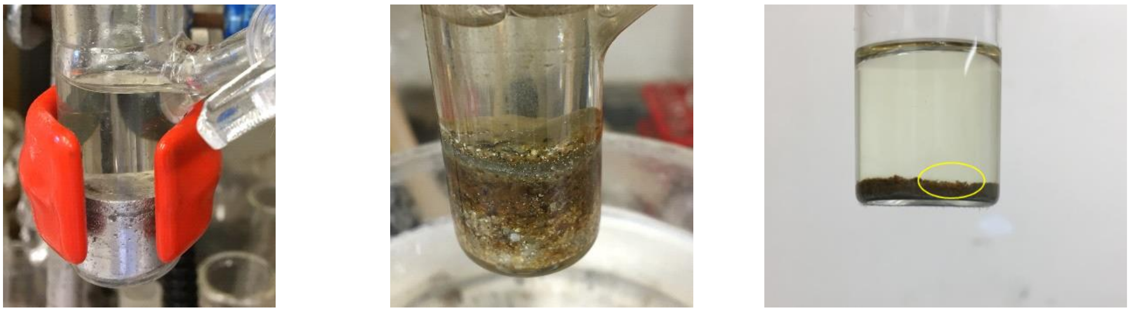

(a) **(b)** **(c)**

Fig. S4-1: **(a)-(b)** Evolution of the reaction media during the reaction of C_2N using mercury/potassium amalgam. **(c)** ~~Crude product of C_2N after amalgam removal~~.

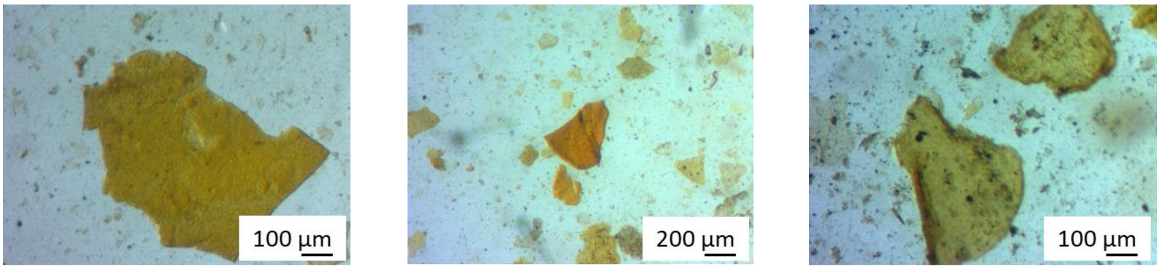

(a) **(b)** **(c)**

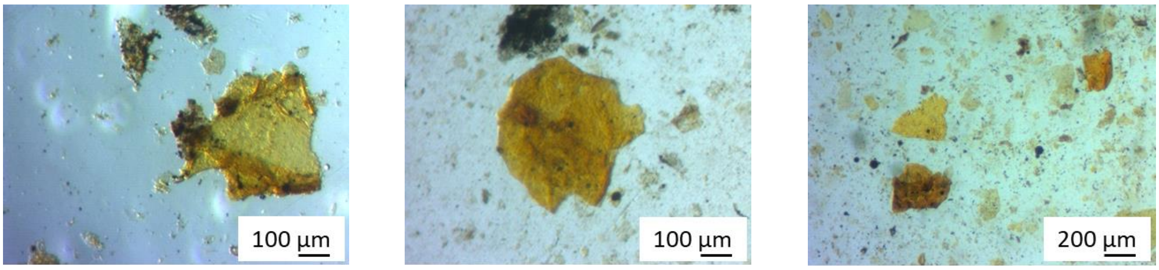

(d) **(e)** **(f)**

Fig. S4-2: Optical microscopy analysis of C_2N obtained using Hg/K amalgam. The flakes were suspended in a 1:2 / EtOH:HCl (v/v) solution.

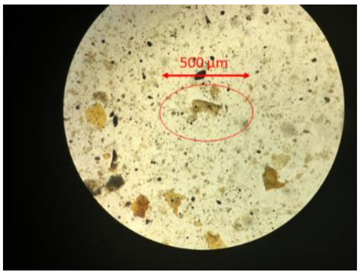

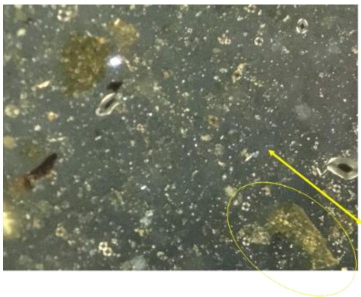

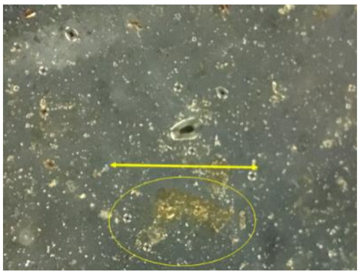

(a) **(b)** **(c)**

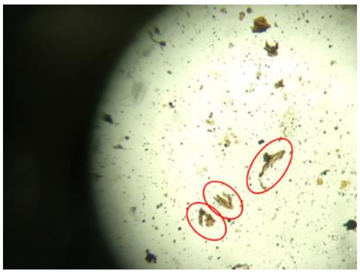

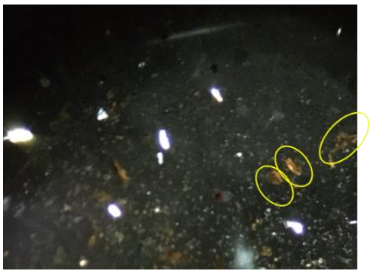

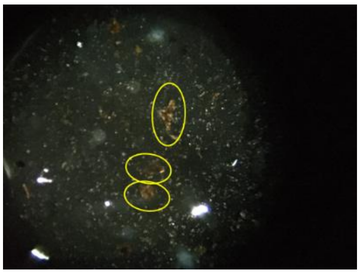

(d) **(e)** **(f)**

Fig. S4-3: **(a)-(c)** Series of polarized light studies of thin C_2N flakes (circles). **(d)-(f)** Series of polarized light studies of aged, folded C_2N flakes (circles).

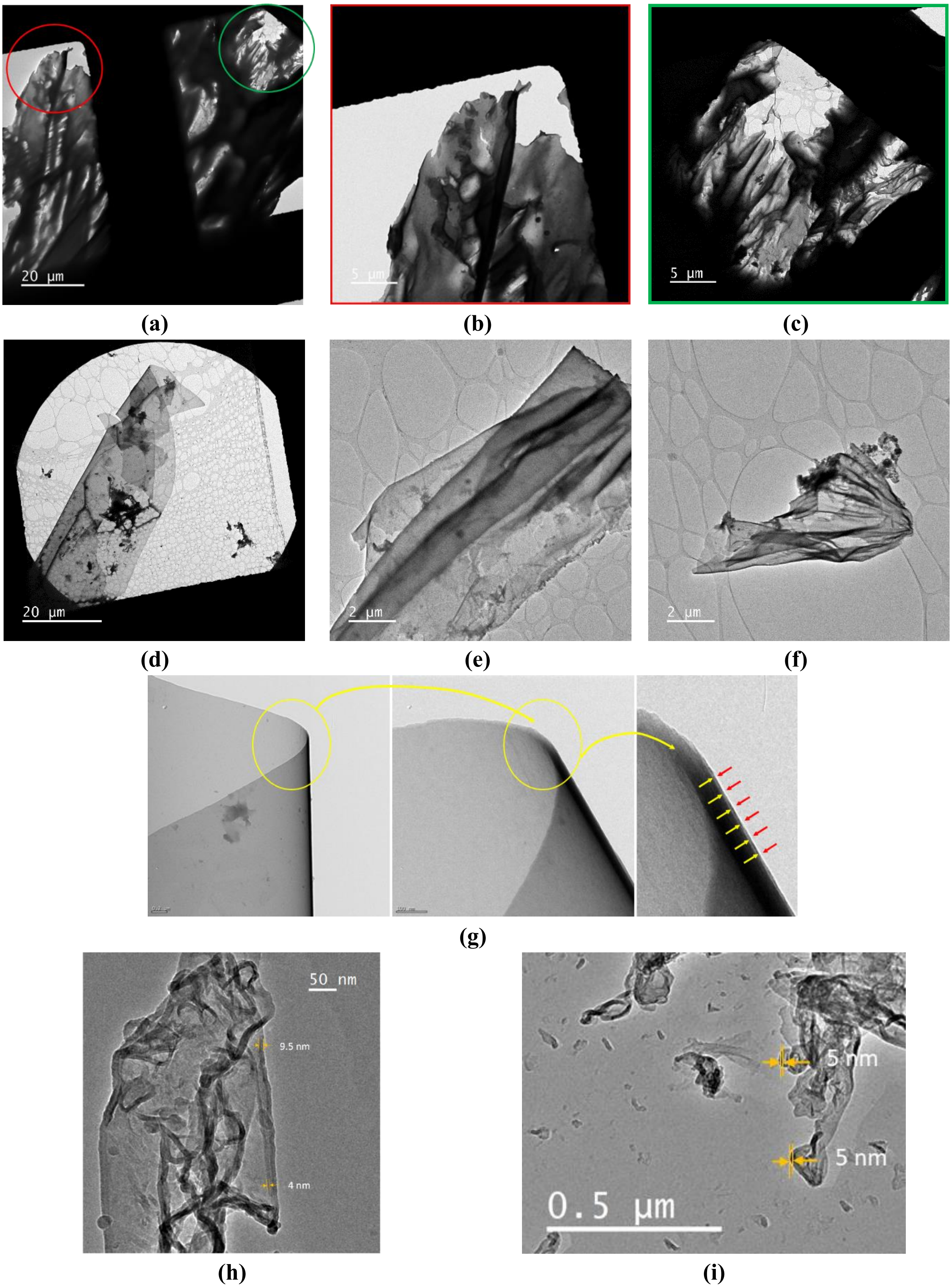

Fig. S4-4: TEM studies of C_2N flake (HCl/EtOH dispersion):
(a)-(f) deposited onto a holey carbon grid (HCl/EtOH dispersion). Variously sized flakes are visible, some of them spanning over several squares. Interestingly, the flake shown on (f) has been pinned by a default on the grid while still being flushed by the dispersion media (acidic water/ethanol) during its deposition on the grid
(g) thick and folded
(h)-(i) at high resolution.
